# Cancer screening simulation models: a state of the art review

**DOI:** 10.1186/s12911-021-01713-5

**Published:** 2021-12-20

**Authors:** Aleksandr Bespalov, Anton Barchuk, Anssi Auvinen, Jaakko Nevalainen

**Affiliations:** 1 Faculty of Social Sciences, Tampere University, Arvo, Box 100, 33014 Tampere, Finland; 2 Petrov National Research Medical Center of Oncology, Leningradskaya 68, Pesochny, Saint-Petersburg, Russia 197758; 3 Institute for Interdisciplinary Health Research, European University, Shpalernaya Ulitsa, 1, Saint-Petersburg, Russia 191187

**Keywords:** Cancer screening, Modelling, Systematic review, Microsimulation, Trends

## Abstract

**Background:**

Nowadays, various simulation approaches for evaluation and decision making in cancer screening can be found in the literature. This paper presents an overview of approaches used to assess screening programs for breast, lung, colorectal, prostate, and cervical cancers. Our main objectives are to describe methodological approaches and trends for different cancer sites and study populations, and to evaluate quality of cancer screening simulation studies.

**Methods:**

A systematic literature search was performed in Medline, Web of Science, and Scopus databases. The search time frame was limited to 1999–2018 and 7101 studies were found. Of them, 621 studies met inclusion criteria, and 587 full-texts were retrieved, with 300 of the studies chosen for analysis. Finally, 263 full texts were used in the analysis (37 were excluded during the analysis). A descriptive and trend analysis of models was performed using a checklist created for the study.

**Results:**

Currently, the most common methodological approaches in modeling cancer screening were individual-level Markov models (34% of the publications) and cohort-level Markov models (41%). The most commonly evaluated cancer types were breast (25%) and colorectal (24%) cancer. Studies on cervical cancer evaluated screening and vaccination (18%) or screening only (13%). Most studies have been conducted for North American (42%) and European (39%) populations. The number of studies with high quality scores increased over time.

**Conclusions:**

Our findings suggest that future directions for cancer screening modelling include individual-level Markov models complemented by screening trial data, and further effort in model validation and data openness.

**Supplementary Information:**

The online version contains supplementary material available at 10.1186/s12911-021-01713-5.

## Background

In 2017–2018, approximately 17 million incident cancer cases (excluding nonmelanoma skin cancer) and 9.6 million cancer deaths occurred annually worldwide [1, 2]. Early detection and screening programs have been suggested for control cancer by the WHO [3, 4]. However, introduction of a cancer screening program requires careful assessment of priorities, health care capacity, and potential impact. Assessment of prerequisites and likely outcomes is needed for decision-making about screening, and simulation models to provide evidence for such policy decisions [5]. Modeling can be especially important in settings where empirical data are absent or unavailable, to adjust screening parameters and define the scope, target, and anticipated impact of screening [6, 7].

Therefore, simulations based on mathematical and statistical models are widely applied in cancer screening [8–10]. They are used for evaluating both the effectiveness and economic impact of cancer screening. Besides, they can complement randomized screening trials by fulfilling the gaps in understanding of population-level screening effects that cannot be obtained from trial data.

The evidence obtained from simulation and empirical studies can currently support the implementation of only a few cancer screening interventions: most of the research is focused on breast, cervical, colorectal, lung, and prostate cancer screening programs. Various cancer screening programs are on-going in several countries: cervical cancer screening with cytology and HPV test [11], mammography screening for breast cancer [12], several modalities of colorectal cancer screening [13]. There is some evidence of the effectiveness of PSA-based prostate cancer screening [14], but large-scale implementation has not been recommended because of the unfavorable balance of benefit and harms [15]. Similarly, there is some evidence for the effectiveness of lung cancer screening with LDCT among heavy smokers [16], but it has not been widely implemented on a population-level program so far.

Existing cancer screening models can be divided into broader types or families based on their principal features. Distinct approaches to simulation have their model assumptions, properties, requirements, and most appropriate applications. This variability of models complicates the assessment of their benefits and shortcomings.

Several systematic reviews [17–19] and comparative analyses [20, 21] of cancer screening simulation studies have appeared recently. However, they have several limitations: they mostly compare model outcomes (usually estimated effects of screening) [22] and build more precise and plausible evaluations of these outputs. This review aims to summarize the 'big picture' of the methodology for cancer screening simulations. We do not identify our research as "Systematic Review" in the PRISMA terminology. However, we incorporate some ideas of "Systematic Reviews," thus we include the PRISMA checklist (Additional file 1). More specifically, our goals within this state of the art review are:

- To describe methodological approaches used in cancer screening simulation;
- To characterize the distribution of the cancer sites for which the models are applied;
- To evaluate the quality of cancer simulation studies and approaches;
- To assess differences in studies approaches across geographical regions;
- To describe trends over time in the previous aspects.

## Methods

### Eligibility criteria

All published studies that described the development or application of a cancer screening model were defined as potentially eligible. Besides, eligible studies shall be focused on lung, breast, cervical, colorectal, or prostate cancer. Individual risk models, animal, cell line, compound, and clinical studies were excluded. Systematic reviews and meta-analysis were used for identifying additional studies. We included studies published from January 1998 to September 2018, in English language. We used Medline, Web of Science and Scopus for the paper search. Reference lists of the systematic reviews on the subject of this paper were also searched for additional papers. All studies in the reference lists of the reviewed publications that were not found by the literature search were manually checked for compliance with the search keywords. New studies thus identified were added to the list of eligible studies for abstract screening.

### Search strategy

Search terms were: "Cancer", "Cancer type", "Simulation", "Simulation modality". Two types of queries were created: (1) for models with one cancer type and one modality, and (2) for models that include at least two modalities or cancer types. The search strategy has been developed in consultation with an informatician at Tampere University. It is described in detail in Additional file 2: Appendix A.

The search criteria for the clinicaltrials.gov database were 'cancer' and 'screening.'

### Searched data filtering

**Fig. 1:**
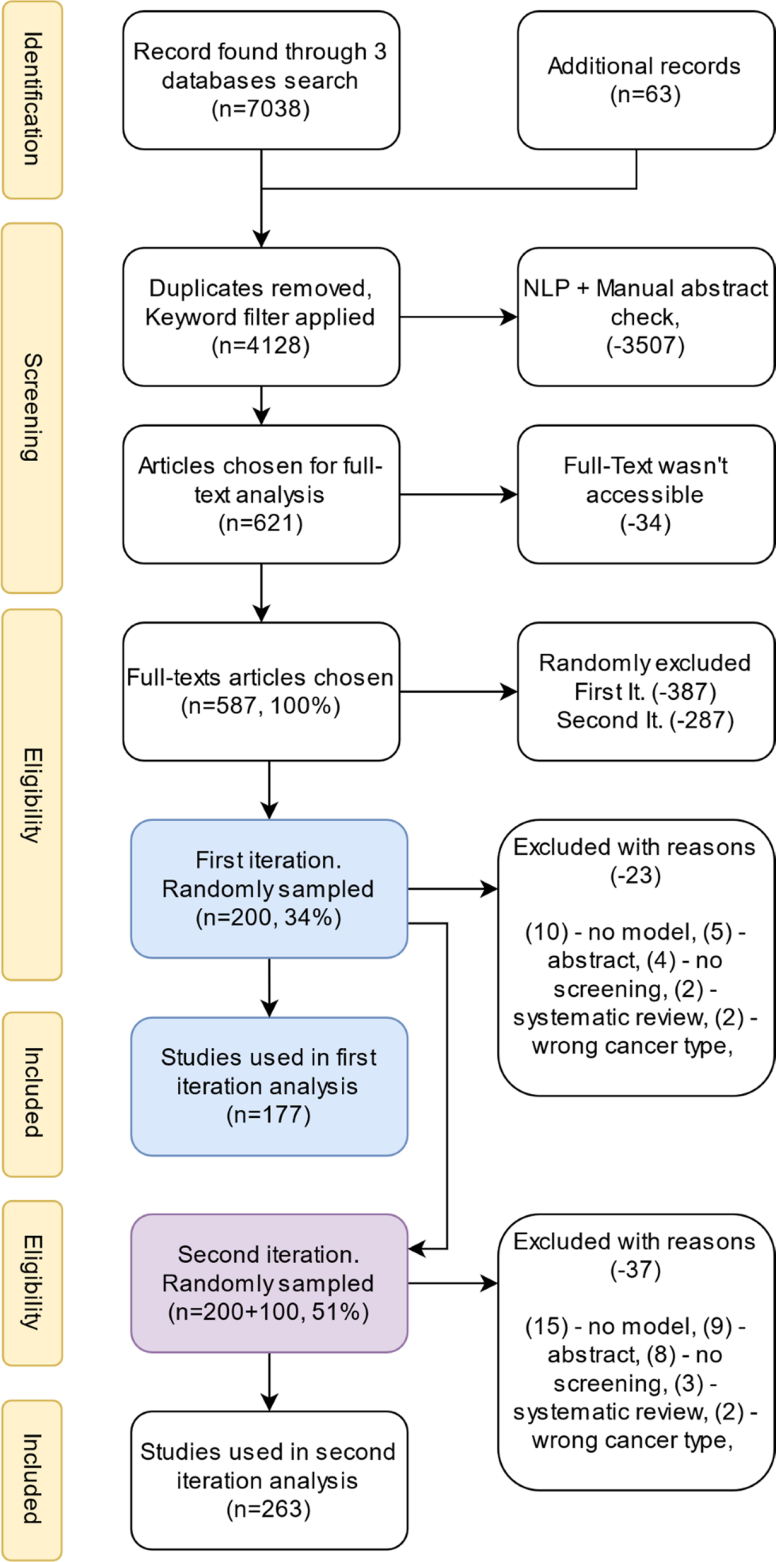
Flow diagram of study selection process

The search results included 2236 records in Medline, 1447 in Web of Science, and 3355 in Scopus (Fig. 1). Further 63 studies were added from the bibliographies of systematic reviews. The retrieved records from all three databases were processed by a Python script to exclude duplicates and filter out inappropriate studies (such as those focusing on image recognition in screening, comparative analysis of screening tests, and radiation-induced health risks in lung cancer screening). Additionally, the script formatted the records to facilitate manual review. After filtering, the dataset comprised 4128 (58% of the initial number) records.

### Abstracts screening

The two first authors independently checked titles and abstracts of the remaining records. The form for manual abstracts check is described in Additional file 2: Appendix C. Papers inconsistent with the eligibility criteria were excluded. As a result, 621 studies were chosen for full-text analysis.

### Full-text processing strategy

A total of 587 full-texts of the 621 studies (94.5%) were retrieved, but we could not access 34 (5.5%) of them. Some papers were behind a paywall for university library subscription, and some had broken links. Evaluating nearly 600 full-text articles turned out to be an overwhelming task, and therefore a random subset of 200 papers was selected for the analysis. This sample was processed and scored by reviewers using a Windows Form and the Pubmed API—"Entrez Programming Utilities" [23].

We also used simple NLP techniques to extract data from the full-text papers. Several classification algorithms like SGDClassifier, Random Forest, SVM were used to classify abstracts as eligible or not during the selection stage. All of them had comparable results with AUC ROC (0.9–0.95) and were used to filter non-eligible records. The final classifier (SGDClassifier) was chosen with a threshold that corresponded to Sensitivity = 1 and maximal possible Specificity = 0.83. Training dataset for the classifier can be found in Additional file 3. However, the classifier alone did not make the decision whether a record was valid or not; the records classified as potentially valid were evaluated by the reviewers.

An automatic keywords extraction algorithm was used during the full-text analysis to obtain the main study concepts and features such as cancer types, model types, and study outputs. A description of the data extraction algorithm can be found in Additional file 2: Appendices B and D. In the case of significant contradictions between the scores of the two reviewers, a third party scored the texts independently.

All data were grouped and stored in CSV tables. Python libraries numpy, scipy and matplotlib were used to extract and visualize the data. The analysis was performed in two iterations (Fig. 1). At the first iteration, 200 full texts were examined, and 177 of them were accepted. To confirm the existing trends or to find contradictions, 100 randomly selected full texts were additionally examined. Finally, 263 full texts were used in the analysis. No substantial differences in the trends between the two iterations were found.

### Definitions for model classification

During the data analysis, we compiled several keywords describing simulation approaches and models used by authors to describe their models. Several entries were combined to simplify the classification and some conceptually similar simulation approaches were merged. The full classification unification table can be found in Additional file 2: Appendix D (4.4). We identified four main simulation approaches that applied to 87% of considered studies.

A cohort-level model (CLM) refers to a Markov chain model used to calculate the transitions from one population group to another.

An individual-level model (ILM) is a Markov chain model that calculates transitions between health states for an individual. The most popular name for such models is microsimulation, but not all authors have consistently used this term [24].

Regression models cover all types of regression (that are not Markov models): e.g., linear models, generalized linear and non-linear models, multivariate models, and mixed-effects models. This group includes all cohort and individual level regressions.

Differential equations (DE) models can also be linear, non-linear, or partial, etc. This group includes all individual and cohort level DEs.

We classified studies as "applied" if the model was used to obtain the study's main result, and "developed" if the paper's main outcome was the model itself, and the model is described in sufficient detail to be reproduced. Studies that could not be classified into one of the two above mentioned categories were labeled as "Other."

### Quality assessments

We evaluated the quality of the conduct and reporting of each article based on the following key elements: if the study included a: validation of the results (V), sensitivity analysis to evaluate model uncertainties or robustness (S), discussion of the limitations of applicability (L), and appropriateness (A).

Appropriateness was defined as the consensus of expert opinions on papers on whether (1) numerical indicators properly summarize the key findings of the study; (2) confidence intervals were available for the key results; and (3) a critical evaluation of strengths and weaknesses of the study was included in the discussion.

Study assessment was divided into two parts: manual and automated assessments. This approach was taken to compensate for experts' possible subjectivity during the assessment and for possible errors. However, an automatic assessment alone may have also missed meaningful features; thus, manual assessment was also necessary.

At first, experts filled in a checklist manually. As a part of the procedure, they assessed the study with an overall mark (from 0 to 5). Then they discussed their assessments and chose a consensus mark for the study.

Secondly, a computer program used experts-filled checklists and keyword search in study text to assess studies automatically. For each study, the program assigned V, S, L, and A criteria (as defined above) with a score of 1 or 0 using the checklist and article text. For instance, if "sensitivity analysis" was not ticked on the checklist, but was found in the keyword search of the text, the program assumed that a "sensitivity analysis" was actually performed. If "sensitivity analysis" was in the checklist, no keywords search was performed. However, the assessment in manual review could have been based on another term like “model quality analysis”. The automated score was calculated as:

If the model was APPLIED and DEVELOPED in the paper:

- SCORE =  + 2 (V) + 1 (S) + 1 (L) + 1 (A);

If the model was just DEVELOPED in the paper without a real application of screening assessment:

- SCORE =  + 2 (V) + 1 (L) + 2 (A);

If the model was just APPLIED in the paper (model was developed elsewhere and considered validated):

- SCORE =  + 2 (S) + 1 (L) + 2 (A).

Finally, the results of the experts' consensus and automatic assessment were averaged to provide the robust, final quality score.

Each paper could receive a score from 0 to 5. Papers were regarded as high-quality if they received a quality rating ≥ 4. Other papers were marked as standard quality. Some parameters of quality assessment were outside the scope of this review. For example, detailed elaboration of sensitivity analysis by types of uncertainty: validity of assumptions, missing data, external validation, or model choice rationale were not evaluated. More information on studies validation procedures can be found in Additional file 4.

## Results

### Basic full-text analysis

#### Which cancer screening is the most simulated?

**Fig. 2:**
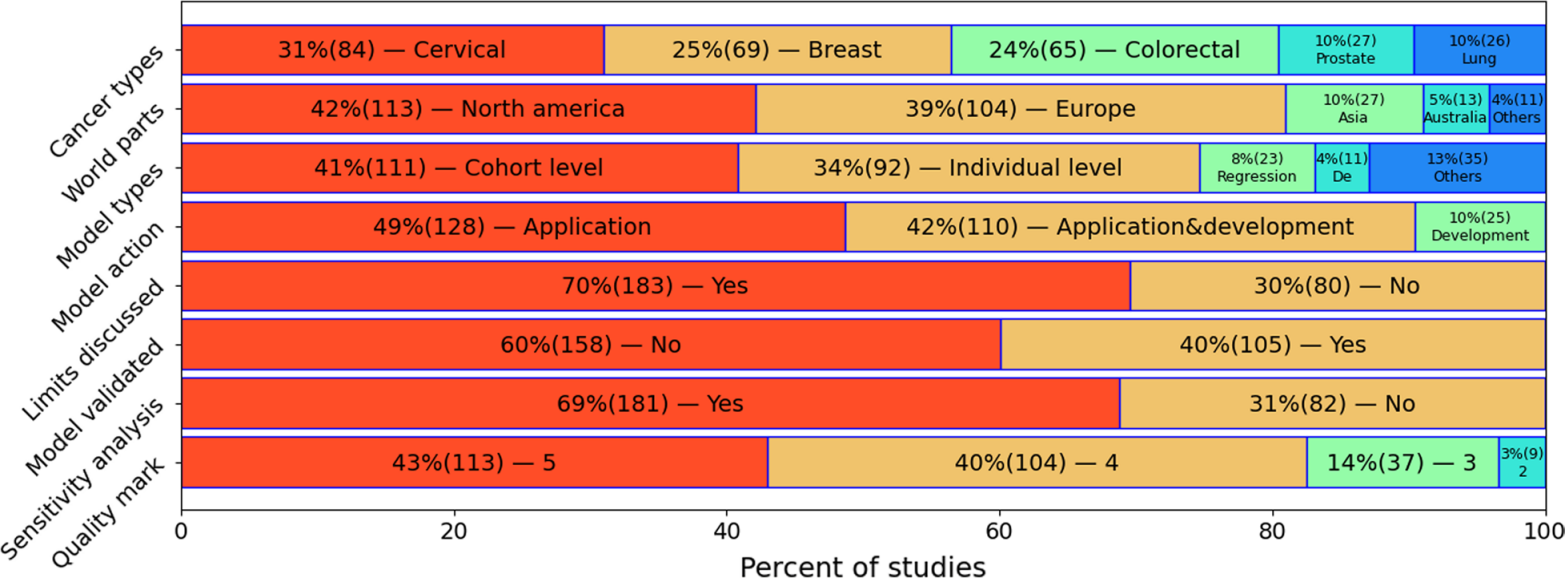
Characteristics of considered studies as a proportion (frequency).”World Parts” = sources for the study populations by region

The most common cancer type in the papers selected for the study was cervical cancer (n = 84, 31% of the total) (Fig. 2). However, most studies addressed cervical cancer prevention and vaccination rather than screening as the main topic (n = 63, 75% of the studies on cervical cancer). Breast cancer was considered in n = 69 (25%) of the studies. Majority of breast cancer studies evaluated mammography screening effectiveness (n = 38, 55% of the breast cancer papers). Several studies focused on breast cancer risk factors as one of the main study questions (n = 16, 23%). Nearly a similar number of studies were related to colorectal cancer (n = 65, 24%). A substantial proportion of these was dedicated to optimizing screening test usage (n = 27, 41% of the colorectal cancer studies), e.g., considering different cut-offs for the iFOBT test [25] and cost-effectiveness of various approaches. In studies of prostate cancer (n = 27, 10% of the total), the most common goal of screening simulation was assessing the effects of introducing prostate-specific antigen PSA-based screening (n = 19, 70%). Only 26 (10% of the total) studies dealt with lung cancer screening simulation, with the efficiency of screening tests the central question in these studies (n = 13, 48% of the paper on this site). The most common objective was evaluating the cost-effectiveness of various screening strategies or approaches (n = 186, 71%).

#### Which type of model is the most popular?

Markov models are the most popular for cancer screening simulations (Table 1). The other types of models were not used even nearly as frequently. The most frequently used model type in cancer screening simulation studies was the CLM (n = 113, 42%). However, of the CLMs on cervical cancer, a large proportion simulated the effectiveness of cervical cancer vaccination rather than cervical cancer screening. If these studies were excluded, individual-level Markov models became the most popular model type in all models (86 ILM, 84 CLM).

**Table 1:** Characteristics of considered approaches (% of all studies)

Characteristic	Individual level	Cohort level	Regression	DE	Other
Breast cancer	25 (10%)	25 (10%)	6 (2%)	2 (1%)	12 (5%)
Cervical cancer (all)	19 (7%)	49 (19%)	8 (3%)	5 (2%)	8 (3%)
Cervical cancer (no prevention)	6 (2%)	21 (8%)	5 (2%)	0 (0%)	4 (2%)
Colorectal cancer	30 (11%)	25 (10%)	5 (2%)	2 (1%)	3 (1%)
Lung cancer	11 (4%)	8 (3%)	0 (0%)	1 (0%)	6 (2%)
Prostate cancer	14 (5%)	5 (2%)	4 (2%)	1 (0%)	6 (2%)
Avg. Quality	4.5	4.1	3.7	4.4	4.1
Application	83 (32%)	105 (40%)	20 (8%)	11 (4%)	26 (10%)
Development	16 (6%)	75 (29%)	17 (6%)	7 (3%)	10%)

Note that the percent sum can be greater than 100%, because all characteristics are not mutually exclusive (e.g. a study on breast and lung cancer that exploits both an ILMs and a CLMs simultaneously)

However, CLMs were most frequently used to simulate cervical cancer screening. Even if prevention studies were excluded, CLMs still dominated with 21 CLMs and 6 ILMs.

We found 23 (8%) models that used different types of regressions as the simulation basis. Even though we decided to consider this group as a single category, it is worth noting that eight regression models dealt with individual level and 15 with cohort level. There were 11(4%) DE models that could be further divided into five individual level and six cohort level models.

#### Quality assessment of the models

Best average scores were observed with studies employing ILMs: 4.5. Most of them (91%) had a score ≥ 4. The lowest average quality studies were those with regression models: 3.7 (56% with score ≥ 4). The average quality score for CLMs studies was 4.1 (81% with score ≥ 4), and for DEs, the average score was 4.4 (81% with score ≥ 4) (Table 1).

#### Regional differences in screening simulation

Most of the included studies had been conducted on North American and European populations (Fig. 2). The North American population was more widely used for colorectal cancer screening simulations (n = 36, 30%), while the studies with the European population focused on the breast (n = 34, 33%) and cervical (n = 33, 32%) cancers.

The European population was rarely used for lung cancer studies (n = 4, 4%). Most of the studies on other populations dealt with cervical cancer screening and prevention simulations: all studies on African or South American, and half of those on Australian and Asian populations. In contrast, of the studies on the North American population, cervical cancer screening was rarely addressed (n = 17, 14%). The distribution of cancer types for different population groups can be found in Additional file 2: Appendix H (8.2).

#### Validation perspective

Most of the studies 77 of 106 (73%) that reported validation used the direct comparison of a specific outcome to external data as a validation scheme. Incidence alone was the main validation outcome in 36 (34%) studies. Mortality alone was used in validation in 13 (12%) studies. Studies were validated by both outcomes in 29 (28%) studies. Several studies assessed the model fit to the internal data of the study 22 (21%) or used cross-validation 21 (20%). Five studies (5%) reported that their model had been validated in earlier studies. Validation data origins were not indicated clearly in 26 (25%) studies.

### Time trends

**Fig. 3:**
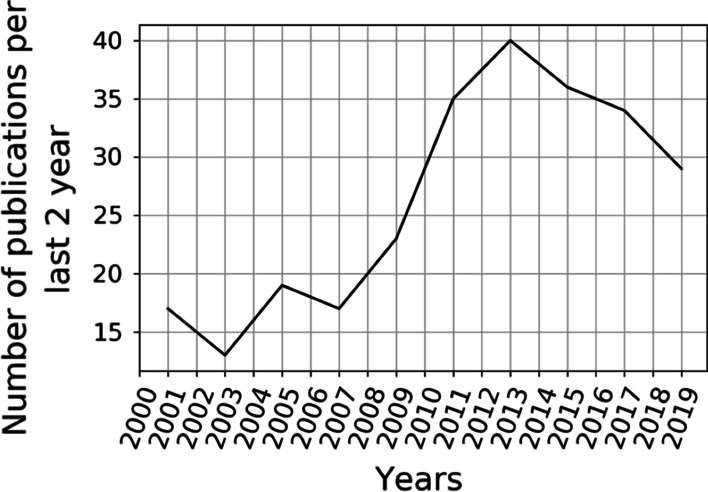
Studies publication dynamics. Every data point represents the number of papers published during the previous 2 years

The increasing publication rate in cancer screening simulation reached its turning point in 2013 (Fig. 3). The trend showed a steady increase from approximately six papers on a topic per year in 2003 to 20 papers per year in 2013. Then the rate started to decrease to 14 papers per year by 2019.

#### Simulation trends

**Fig. 4:**
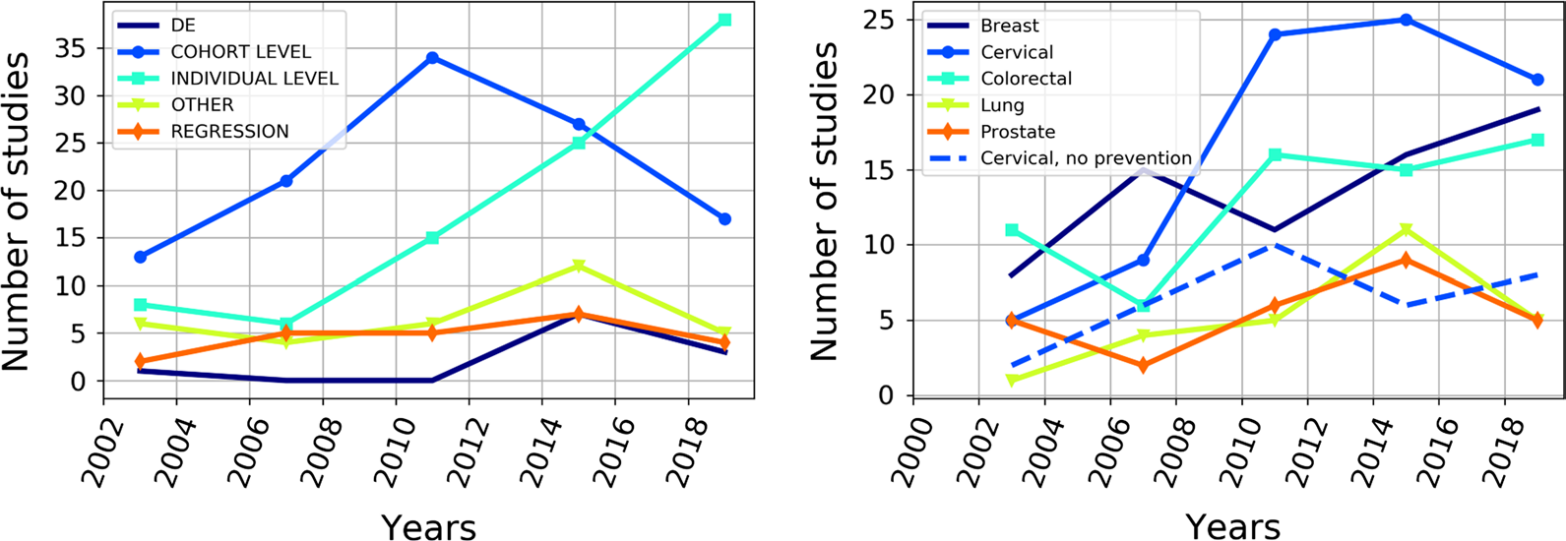
Trends in the interest to the screening of different cancer types and model types. Every point is the number of papers published during the previous 4 years. **a** Studies stratified by model type. **b** Studies stratified by cancer type

Publication counts of the studies stratified by the model type show that CLMs were used most frequently around 2010 (Fig. 4a). More than 30 studies exploiting this simulation approach were published during 2007–2011, and subsequently, their number has decreased. Simultaneously, the number of publications based on ILMs increased swiftly. Moreover, the number of studies with other models than ILM and CLM decreased after 2015. These dynamics appear to relate to the increase in ILMs because the peak in the use of "other models" also occurred during the CLM models trend peak.

#### Quality trends

**Fig. 5:**
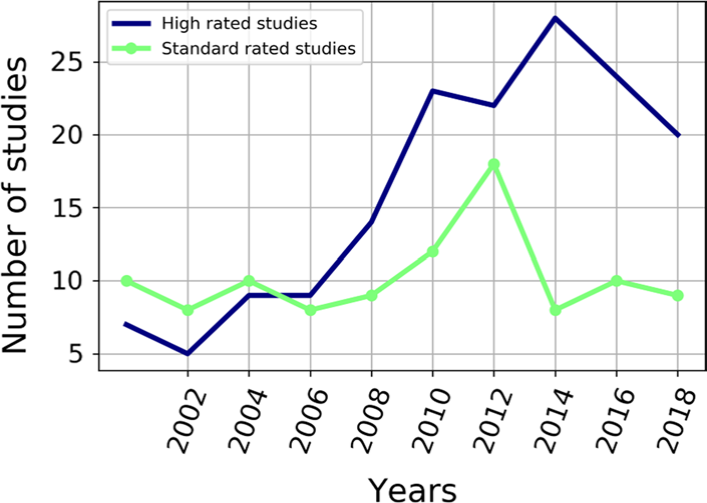
Quality of studies over time. Every data point represents the number of high and standard rated studies for papers published during the previous 2 years

A rather optimistic picture emerges on a scale of the last 20 years. Overall, more than 80% of considered publications can be called "high-level" (Fig. 2). The odds of high versus standard quality score publications reached 2.5 by 2018 (Fig. 5). This reflected a relatively constant number of publications with lower scores, while the number of those with high-quality scores has increased. Also, there were some differences between the manual and automatic quality assessment: the mean score of manual assessment was 3.46 (SD 1.02), whereas the automatic assessment gave a mean of 2.71 (SD 1.42). Pearson correlation coefficient between them was 0.95. Thus, manual estimation assigned higher average scores, but both put them in the same order.

#### Cancer trends

The results of the stratification of the found publications by type of cancer are shown in Fig. 4b.

Colorectal cancer and breast cancer were the most common sites evaluated in modeling studies and show increasing trends, while analyses focusing on lung cancer and prostate cancer were substantially less common topics. However, they also show an increasing trend. The cervical cancer screening trend reached saturation in 2010.

## Discussion

### Results of the evaluation

In this review, we systematically described trends in methods and topics in simulation studies of cancer screening. We analyzed trends in the distribution of specific cancer types, geographic regions, and various simulation approaches.

The quality of studies, evaluated by four simple criteria, has steadily increased over the past 20 years. Some previous systematic reviews have assumed that study quality was constant over time, but such assessment has been based on a small number of studies used for a trend analysis [26]. We were able to score > 250 studies using consistent criteria. The number of publications graded as high-quality studies increased steadily. The trend toward increasing quality may reflect more stringent requirements for publication. However, other systematic reviews have indicated significant shortcomings: most studies had failed to incorporate sensitivity analysis by types of uncertainty, external validation, and model choice rationale. Such shortcomings also appear in new studies [27].

Among the reviewed studies, the effectiveness of the screening of colorectal cancer was the most frequent topic. The trends in the reviewed studies show the growing breast cancer screening priority. Perhaps the effectiveness of mammography screening has already been established. The main task is the practical implementation of screening programs [28]. According to our results, the most important issue in cervical cancer simulation was not screening per se, but the comparison of screening effectiveness vs. vaccination (prevention). Thus, the number of simulations on cervical cancer screening is decreasing. This indicates that the prevention, rather than screening is the main priority for cervical cancer [29]. Most of the simulation studies (88%) focused on vaccination and screening cost-effectiveness with the conclusion that prevention can achieve a larger population impact. Our estimate of the cervical cancer studies' scope is in good agreement with the previous review, where 84% of all considered studies were focused on cost-effectiveness [26].

According to the observed studies lung cancer screening programs show contradictory dynamics. The likely reasons for that are the previous difficulties in developing successful cancer screening programs [21], and at the same time: rapid development in lung cancer screening [30], the development of personalized screening [31], and the success of the latest trials and pilot programs [32].

Prostate cancer remains a low priority in terms of the scientific community's attention, which likely reflects the low mortality impact of the recent PSA-based screening trials, with substantial overdiagnosis offsetting the potential benefit [15]. New screening modalities have not yet reached the stage where their applicability in screening would be evaluated on a large scale, although magnetic resonance imaging appears to reduce overdiagnosis substantially [33].

Most studies conducted for low-income countries' populations address cervical cancer vaccination and do not deal with screening of any other type of cancer. This likely reflects the limited feasibility of screening in the setting of low-income countries due to a lack of adequate cancer registries, health care organizations, and financial constraints [34].

The most popular approaches in modeling were the two types of Markov models. Over the past ten years, an initial increase in CLM's popularity due to its simplicity was followed by a decline. It is possible that such development was probably owing to the limits of the approach, such as its inability to follow more detailed dynamics within the population, the difficulty in reuse and problems with the quality of research. The transition from CLM to ILM was accompanied by a general improvement in the quality of published studies. This likely relates to the fact that new ILMs are not created very often, and once validated, well-established ILMs are subsequently used extensively. Thus, they do not face validation issues from which all new models suffer. Adapting an existing ILM is an attractive alternative to the development of a new model. However, this situation cannot be regarded as fully satisfying. The most popular microsimulation models were created by large institutes and are not open source and freely available. Limited access to individual-level data used for ILM fitting also limits the transparent evaluation of the methods. Initiatives for open data may solve these problems in the future, but privacy issues and reuse conditions have not been resolved with sensitive health information. Moreover, the dominance of a single approach would lead to reduced diversity of methodological approaches, which could restrict perspectives.

### Study limitations

Our study has some important limitations. First, we searched the literature only for publications in English, which can affect the geographical distribution of the studies and indirectly influence other findings. This could occur if, for instance, certain cancer types are a prominent focus in areas favoring English in research.

The search strategy was created to capture studies covered by the search terms listed in “Search strategy” section. We obtained our study material via a systematic search of publication databases. The search is limited to the coverage of the three publication databases searched. We could have missed some less frequently used synonyms for the terms.

We analyzed the random subset of 300 out of the 587 studies and finally included 263 fulfilling the eligibility criteria. The two-step analysis was chosen to improve the representativeness and validity of the results. Even though we expect that the subset was representative and used a two-step analysis to ensure consistent results, the full set could have provided more insight into the matter.

Another limitation is that our quality assessment method has not been validated, or was not based on the “delphi” method or other standard procedures. Nevertheless, we believe that the two aspects of analysis used as quality indicators (validation and sensitivity analysis), and two items pertaining to reporting (appropriateness and limitations), reflect quality of the study conduct and reporting similarly as in well-established quality assessment tools.

The automated quality assessment showed a good overall correspondence with the manual assessment. The limitation of both (their averaging) is that they are based only on the presence of a specific study component (e.g. "sensitivity analysis"). We did not assess how or how well it was conducted. Some claims of sensitivity analyses (or similar) may thus not be warranted. However, as all the papers are peer-reviewed, we believe that at least minimal criteria have been achieved.

Also, our quality assessments were limited in scope and might not be able to separate shortcomings in reporting from those in study conduct. This could either overestimate or underestimate the proportion of high-quality studies. The reason for not including some of the assessment parameters is that previous systematic reviews showed a disappointing absence in the completeness of reporting all relevant features for colorectal and cervical cancer [19].

Finally, we concentrated on topics and methodological choices of published articles, and our assessment does not cover issues such as overdiagnosis, false positive screening results, complications or costs even though these can be important for decision making.

## Conclusions

To conclude, we have reviewed the most recent results of cancer screening approaches across the world. We introduced the studies' classification based on model type, cancer site, target population country, the model used, and study quality parameters. The analysis incorporated 263 full-texts found through a systematic search of three publication databases from 1998 to 2018. Our analysis shows that currently, the most commonly used approaches to modeling cancer screening are ILMs (34%) and CLMs (41%). ILMs have become the most used simulation approach over the past 5 years and eventually surpassed cohort-level models that were more used previously. The proportion of studies with high-quality scores increased over time.

At present, breast cancer and colorectal cancer are the most common sites evaluated in cancer screening simulation, each representing about a quarter of all studies. The number of studies on these cancer types increased 1.5–1.7 fold during the study period. Most of the cervical cancer simulation studies deal with prevention assessment as the primary study goal. Only 17% of the modeling studies addressed cervical cancer screening (outside vaccination).

Most assessed studies have been conducted for North American (42%) and European (39%) populations. The main focus of interest in studies of the North American population was colorectal cancer (30%), and for Europe, breast (33%) and cervical (32%) cancers.

The trends suggest that cancer screening studies will extensively use individual-level Markov models. Further effort will be needed in profound model validation, code availability and data openness.

## Supplementary Information

**Additional file 1.** PRISMA checklist.

**Additional file 2.** Appendix for the article.

**Additional file 3.** Classifier training data.

**Additional file 4.** Detailed information on validation.

## Data Availability

The list of the analyzed publications is available in the: https://github.com/Magisterbes/BespalovPhd/tree/master/Review%20Search. Also, it is included in supplementary information files. Classifier reproduction code: https://github.com/Magisterbes/StudiesClassificationReproduction. Classifier training data and list of analyzed studies can be found in supplementary material.

## Abbreviations

API: Application programming interface
CISNET: The Cancer Intervention and Surveillance Modeling Network
CLM: Cohort level model
CRC-SPIN: The colorectal cancer simulated population model for incidence and natural history
D.Eq., DE: Differential equations
ILM: Individual level model
MISCAN: MIcrosimulation SCreening Analysis (model)
NLP: Natural language processing
PSA: Prostate-specific antigen
RTC: Randomized controlled trial
iFOBT: Immunochemical fecal occult blood test
