# Supplementary material for: Cancer screening simulation models: a state of the art review
Source: BMC Med Inform Decis Mak. 2021 Dec 20;21:359. doi: 10.1186/s12911-021-01713-5 (PMC8690438; doi:10.1186/s12911-021-01713-5)
Supplement: Supplementary file 2 — Additional file 2. Appendix for the article. [file 12911_2021_1713_MOESM2_ESM.pdf]

1 Appendix A. Search strategy

1.1 Search queries basic elements:

1. Basic parts:
   (a) Cancer
   (b) Simulation
2. Modality perspective:
   (a) Screening
   (b) Treatment or therapy
   (c) Prevention
3. Cancer types:
   (a) Lung
   (b) Cervical
   (c) Breast
   (d) Colorectal
   (e) Prostate

1.2 Idea

Type 1 queries: We have to ensure, that published model has at least (two modalities with any cancer type) Or (two cancer types inside with any modality). With this type of queries we'll get models with broad functionality.

Type 2 queries: Published models with narrow functionality. Query with one modality and one cancer type. Then we exclude from Group 2 everything that is in Group 1.

1.3 Terms

Main Terms in Pubmed syntax:

1. ("Neoplasms"[Mesh Terms:exp] OR "Cancer"[tw] OR "neoplasm"[tw] OR "tumor"[tw] OR "tumour"[tw] OR "carcinoma"[tw])
2. ("Artificial Intelligence"[Mesh Terms:exp] OR "Nonlinear Dynamics"[Mesh Terms:exp] OR "Models, Statistical"[Mesh Terms:exp] OR "Markov Chains"[Mesh Terms] OR "Numerical Analysis, Computer-Assisted"[Mesh Terms] OR "Simulation" OR "markov" OR microsimul*)

Modality terms:

3. ("Primary Prevention"[Mesh Terms:exp] OR "prevention" or "vaccination" OR "Vaccination"[Mesh Terms:exp])
4. ("Early Detection of Cancer"[Mesh Terms:exp] OR screen* OR "early detection")
5. ("Combined Modality Therapy"[Mesh Terms:exp] or "chemotherapy" or "radiotherapy" or "irradiation" or "hormon*" or "adjuvant" or "neoadjuvant" or "surgery" or "resection")

Cancer type terms:

6. ("Breast Neoplasms"[Mesh Terms:exp] OR "breast" OR mammogr* OR "Mammography"[Mesh Terms:exp])
7. ("Prostatic Neoplasms"[Mesh Terms:exp] OR prostat* OR "psa" OR "Prostate-Specific Antigen"[Mesh Terms:exp])
8. ("Uterine Cervical Neoplasms"[Mesh Terms:exp] OR cervi* OR "hpv" OR papillomavir* OR "Papanicolaou Test"[Mesh Terms:exp] OR "Human Papillomavirus DNA Tests"[Mesh Terms:exp] OR "colposcopy" OR "Colposcopy"[Mesh Terms:exp])
9. ("Lung Neoplasms"[Mesh Terms:exp] OR "lung" OR pulmon* OR pneumon* OR "LDCT")

10. ("Colorectal Neoplasms"[Mesh Terms:exp] OR "colorectal" OR colon* OR "crc" OR rect* OR "occult" OR sigmo* "Occult Blood"[Mesh Terms:exp]),

NOT terms:

11. NOT "Radiographic Image Interpretation, Computer-Assisted"[Mesh Terms] NOT "Clinical Trials as Topic"[Mesh Terms] NOT "Diagnostic Tests, Routine"[Mesh Terms] NOT "Clinical Trial"[pt] NOT "Randomized Controlled Trial"[pt] NOT "Comparative Study"[pt] NOT "Physiological Phenomena"[Mesh Terms:exp] NOT "Cell Physiological Phenomena"[Mesh Terms:exp] NOT "Randomized Controlled Trials as Topic"[Mesh Terms] NOT "Logistic Models"[Mesh Terms] NOT "Robotics"[Mesh Terms:exp] NOT "Image Processing, Computer-Assisted"[Mesh Terms:exp] NOT "genetics"[sh] NOT "diagnostic imaging"[sh] NOT "metabolism"[sh] NOT "Controlled Clinical Trial"[pt] NOT "Guideline"[pt] NOT "Randomized Controlled Trial"[pt] NOT "Case Reports"[pt]

1.4 Queries type 1

Outer groups query
1 AND 2 AND 4 AND Y; (Y in [6, 10])

Example:
1 AND 2 AND 4 AND 6

1.5 Queries type 2

Cancer type variation query
1 AND 2 AND (3 OR 4 OR 5) AND ((6 AND 7) OR (6 AND 8) OR (6 AND 9) OR (6 AND 10) OR (7 AND 8) OR (7 AND 9) OR (7 AND 10) OR (8 AND 9) OR (8 AND 10) OR (9 AND 10))

QUERY DECOMPOSED IN 30 QUERIES:

1 AND 2 AND (X) AND (Y AND Z); X in [3, 5], Y in [6, 9], Z in [7, 10]

Example:
1 AND 2 AND 4 AND (6 AND 7)

Modality variation
1 AND 2 AND ((3 AND 4) OR (3 AND 5) OR (4 AND 5)) AND (6 OR 7 OR 8 OR 9 OR 10)

QUERY DECOMPOSED IN 15 QUERIES:

1 AND 2 AND (X AND Y) AND Z; X in [3, 4], Y in [4, 5], Z in [6, 10]

Example:
1 AND 2 AND (4 AND 5) AND 6

1.6 WOS and Scopus search remarks

WOS and Scopus do not deal with "Mesh Terms", thus, all this terms and their synonyms were searched in titles, abstracts and keywords or excluded if they were already included for general search in query.

2 Appendix B. Additional Automatic Filtering

2.1 Wos additional refinements

Refined by: PUBLICATION YEARS: (2018 OR 2010 OR 2002 OR 2017 OR 2009 OR 2001 OR 2016 OR 2008 OR 2000 OR 2015 OR 2007 OR 1999 OR 2014 OR 2006 OR 1998 OR 2013 OR 2005 OR 2012 OR 2004 OR 2011 OR 2003) AND WEB OF SCIENCE CATEGORIES: (ONCOLOGY OR PUBLIC ENVIRONMENTAL OCCUPATIONAL HEALTH OR HEALTH CARE SCIENCES SERVICES OR MEDICAL INFORMATICS OR STATISTICS PROBABILITY OR HEALTH POLICY SERVICES OR COMPUTER SCIENCE INTERDISCIPLINARY APPLICATIONS)

2.2 List of terms for automatic filtering (if abstract contains more than 5 uses of any of this terms)

receptors, inhibitors, pharmacophore, compound, virtual screen, derivativ, prolifer, apoptosis, electromagnetic, antenna, breast phantom, antagonists, kinase, nanocomposite, methylthiophene, phosphate, sequenc, spectroscopy, optic, laser, radio, radia, wavelength, nucleotide, microwave, thermoacoustic, interferogram, 3d, geometrical, image, imaging, gene, dose, guidelin, computer-aided, metabolism, peptide, dosimetr, catalytic, robotics, dementia, fibrobla, stroke, high-dose, low-dose, high dose, low dose, pharmacokin, concentrat, ionizat

2.3 NLP record filtering

Procedure:

1. At first 470 records were checked manually to create learning sample:
   - Abstract
   - Title
   - Keywords
   - Authors

   (126 of them were manually evaluated as eligible.)

2. For every record:
   (a) All text information was combines in one text and stemmed
   (b) Term Frequency vectorization was applied
   (c) Most frequent and less frequent terms were excluded from vectorization. Parameters of boundaries were optimized to get best result on test sample.

3. Resulted vectors were divided randomly to learning and test sample. Learning sample - 298, testing - 172.

4. Several classification algorithms like (SGDClassifier, Random Forest, SVM,) were applied to classify records. All of them has approximately same results with AUC ROC (0.9-0.95).

5. The final classifier was chosen with Sensitivity = 1 and maximal possible Specificity.

6. The resulted classifier will be applied to the rest records.

3 Appendix C. Form for manual abstracts inspection

Keywords are automatically highlighted in the abstracts and some fields are also automatically filled.

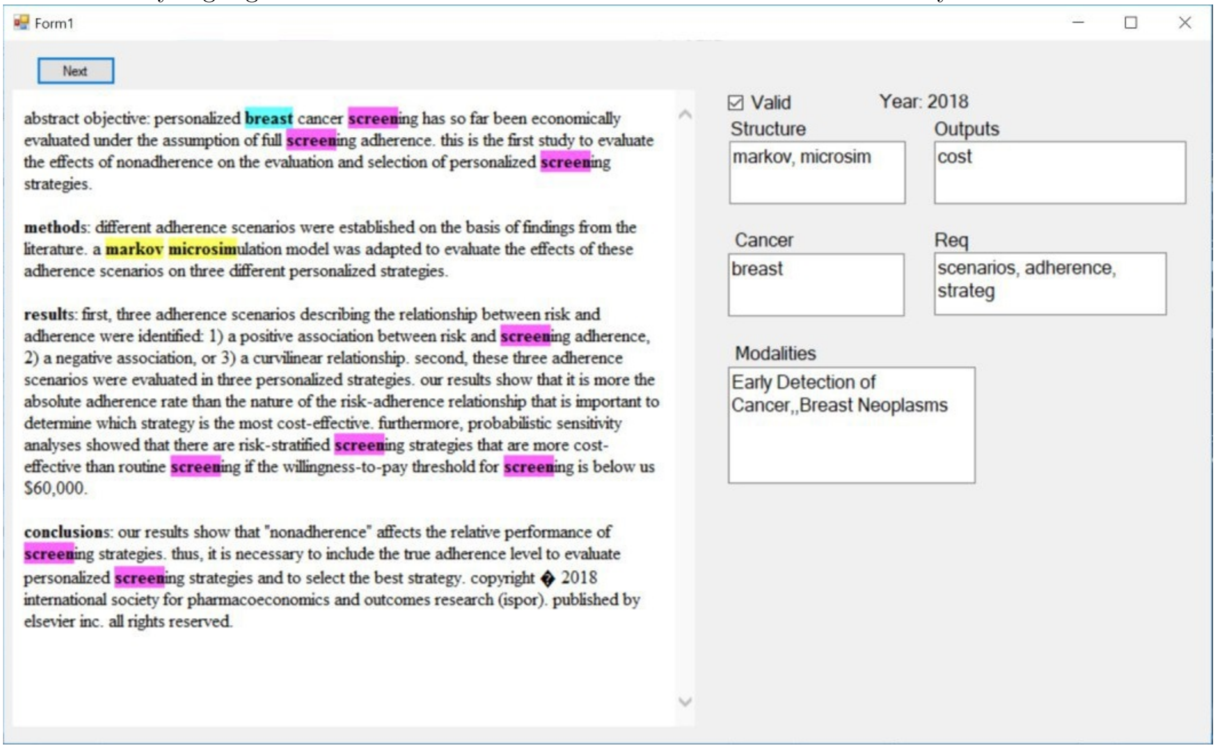

Form fields description

Field Name	Field Description
Valid	Expert evaluation of article abstract suitability
Year	Year of publication
Structure	Short description of the model exploited in the paper
Cancer	Cancer type(s) observed in the paper
Outputs	Measured (calculated) values that presented as the paper scientific results
Req	Keywords that determine the approach of the described research
Modalities	Modality of the research and simulation (early detection, prevention, therapy)

4 Appendix D. Full-text paper evaluation checklist

4.1 Motivation for quality criteria

We have used a Full-text paper evaluation checklist to assess the quality of studies. Our checklist was based on the general criteria commonly used in systematic reviews. However, the features evaluated in systematic reviews are highly dependent on the nature of the research question. Also, they have to be focused on the specific studied outcomes and everything else must be filtered. We were unable to find any previously developed quality assessment tools that would be applicable for our assessment which is not bound to the specific outcomes.

The NOS was developed for empirical studies with individual level data and it cannot be applied in our paper. The items of the scale are simply not applicable. Cochrane RoB and GRADE as well as ROBANS, ORBIT and AHRQ are also reasonably similar. The closest one, due to our knowledge, was PROBAST but is also intended for assessing studies aimed at developing models for clinical use based on empirical data, i.e. not similar to our scope.

We have to admit that our assessment method has not been validated or created by means of a “delphi” method or other highly elaborated procedures. Nevertheless, we can claim that the two aspects of analysis used as quality indicators (validation and sensitivity analysis), and two items pertaining to reporting (appropriateness and limitations), reflect important features in studies, and represent similar constructs (quality of the study conduct and reporting) as in well-established quality assessment tools. We have also added discussion on this aspect in the limitations of the study.

4.2 Automatic part

Automatic data was obtained though the https://eutils.ncbi.nlm.nih.gov/entrez/ service and from the parsed paper pdfs.

Field	Source
Paper Name	Entrez
Paper Authors(All)	Entrez
Authors Affiliations(All)	Entrez
Year of publication	Entrez
Journal	Entrez
Mentioned countries	Paper text
Authors Affiliations(All)	Entrez
Cancer type	Paper text
Model type	Paper text
Sensitivity analysis	Paper text
Analysis outputs	Paper text

4.2.1 Cancer Type keywords

Keywords are counted and number of terms related to specific cancer type compared to the threshold. If the threshold is exceed, it’s considered that the paper is about this cancer type.

1. ”cervical”/”cervix uteri”/”cervix”/”hpv”
   (a) ”cytology” or ”pap” or ”Papanicolaou” or ”smear”
   (b) ”colposcopy”
   (c) ”vaccine” or ”vaccination” or ”valent”
   (d) ”HPV test” or ”digene” or ”cobas” or ”pcr”
2. ”colorectal” or ”bowel” or ”colon” or ”rectal” or ”sigmoid” or ”polyp” or ”adenoma”
   (a) ”fobt” or ”ifobt” or ”fit” or ”feacal” or ”occult blood” or ”Hemoccult”
   (b) ”sigmoscopy”
   (c) ”colonoscopy”
3. ”breast”, ”DCIS”, ”carcinoma in situ”

(a) ”mammorgaph*”
(b) ”ultrasound”
(c) ”clinical breast” or ”CBE”
(d) ”self-breast” or ”self breat” or ”SBE”

4. ”lung” or ”trachea” or ”bronch*”

   (a) ”x-ray”or ”ldct” or ”tomography” or ”low-dose” or ”low dose”

5. ”prostate”

   (a) ”mri” or ”tomograph*”
   (b) ”psa” or ”prostate-specific” or ”antigen”
   (c) ”biopsy”

4.2.2 Model type keywords

Keyword	Term for analysis
MISCAN	MISCAN
CISNET	CISNET Models
regression	regression
differential eq	differential eq
macrosim	macrosimulation
bias model	censoring bias model
fuzzy	fuzzy model
stochastic simulation model	stochastic simulation model
discrete event	discrete event simulation model
discrete-event	discrete event simulation model
survival model	survival model
life-table	survival model
life table	survival model
survival analysis	survival model
microsim	microsimulation
cohort simulation	cohort simulation
hidden markov	hidden markov
hidden-markov	hidden markov
markov	markov model
monte	Monte Carlo model
decision tree	Decision Tree model
decision model	Decision Tree model
decision analytic model	decision model
decision analytic	decision model
decision-analysis	decision model
decision analysis	decision model

4.2.3 Output keywords

Output keywords are extracted and stored.

1. ”life-years” or ”LY” or ”DALY” or ”disability adjusted”
2. ”quality-adjusted” or ”qaly”
3. ”cost-effectiveness”
4. ”cost-utility”
5. ”cost-benefit”
6. ”cost” c,d,e

4.3 Manual part

Windows form was used to extract specific data, which can't be extracted automatically or must be checked

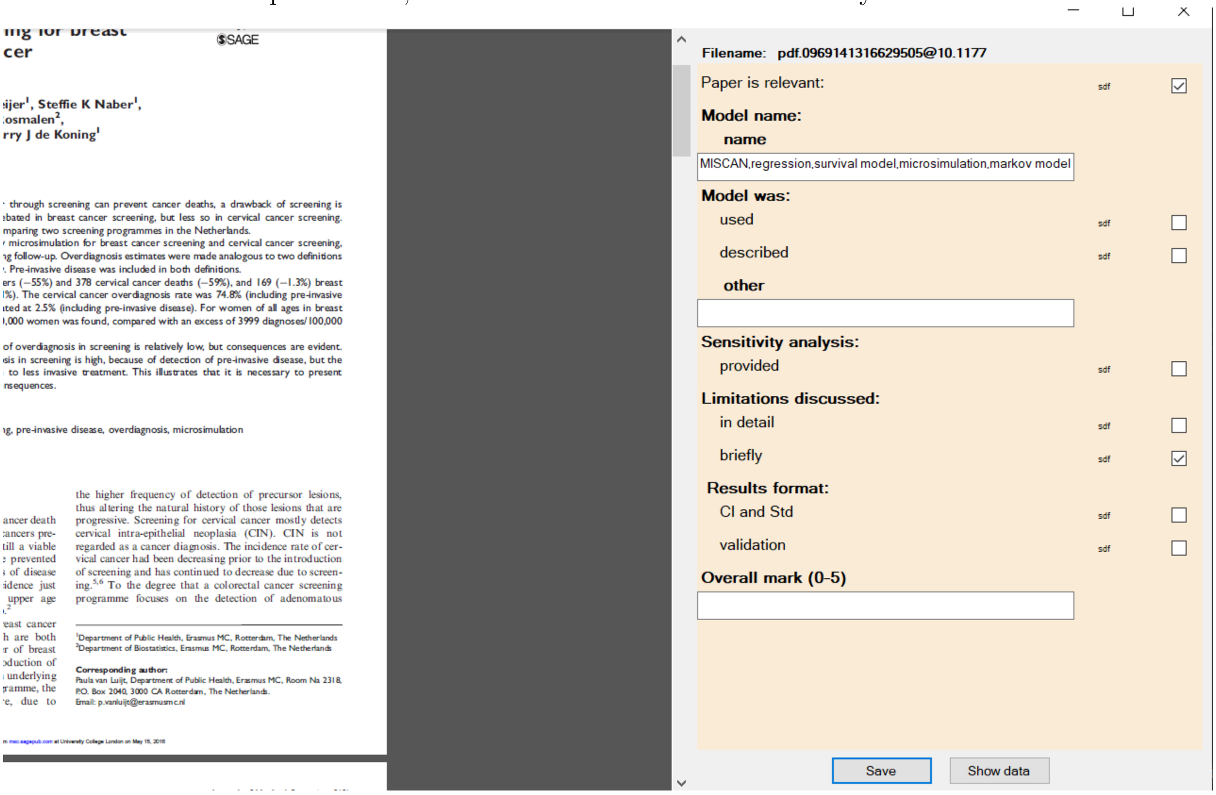

Form fields description

Field Name	Field Description
Paper is relevant	Expert evaluation of the article suitability
Model name	Keywords that shortly describe simulation approach
Model was applied	Main result of the paper obtained from model simulation
Model was developed	Main result of the paper is the model itself. Model described in details so it can be reproduced from this paper.
Sensitivity analysis	Sensitivity analysis and its results are discussed in the paper
Limitations discussed briefly	There are several sentences in the paper about model limitations
Limitations discussed in details	There is highlighted section in which limitations are discussed.
CI and Std	Were the results in the paper were presented in the appropriate format?
Validation	Were the results of model validation presented in the paper?
Overall mark (0-5)	Expert opinion on the paper consistency

4.4 Expert Mark vs. Automatic Mark

The papers are assessed not only by experts. Paper quality is also assessed by algorithm which used less subjective assessment. The results of assessments are combined. Here are the description of the automatic assessment procedure.

1. If model was APPLIED and DEVELOPED in paper:
   (a) +2 model was validated
   (b) +1 there were sensitivity analysis
   (c) +1 Limitations were discussed
   (d) +1 Results were presented in the appropriate format
2. If model was just DEVELOPED in paper:
   (a) +2 model was validated
   (b) +1 Limitations were discussed
   (c) +2 Results were presented in the appropriate format
   !!! screening assessments are not that important if main result is the model itself.
3. If model was just APPLIED in paper:
   (a) +2 there were sensitivity analysis
   (b) +1 Limitations were discussed
   (c) +2 Results were presented in the appropriate format
   !!! model is considered validated.

4.5 Models naming

Authors can give unique names to their models. We've tried to unify model names using keywords which was mentioned in the descriptions of the models. This is our renaming table.

Authors model naming	Model unified renaming
markov model	cohort level markov model
the lung cancer policy model	cohort level markov model
discrete event simulation model	cohort level markov model
excel-based model	cohort level markov model
Smoking-Lung Cancer Model	cohort level markov model

contingent valuation method (cvm)	contingent valuation method (cvm)
differential eq	D.Eq.
hpv dynamic mathematical model	D.Eq.
differential equations	D.Eq.
Dana-Farber	D.Eq.
SCANS (Michigan)	D.Eq.
decision tree analysis	decision tree model
decision tree model	decision tree model
estimation method	estimation method
bayesian hierarchical model	estimation method
MISCAN	individual level markov model
microsimulation	individual level markov model
fhcrc model	individual level markov model
MISCAN PROSTATE	individual level markov model
MISCAN FADIA	individual level markov model
wisconsin breast cancer model	individual level markov model
simcrc	individual level markov model
prohta simulation	individual level markov model
china lung cancer policy model	individual level markov model
cervivac model	individual level markov model
policy1-cervix, hpv-advise	individual level markov model
CRC-SPIN	individual level markov model
UMN-Cervical	individual level markov model
BCOS	individual level markov model
Harvard cervical cancer model	individual level markov model
LCPM (MGH)	individual level markov model
LCOS	individual level markov model
PSAPC (FHCRC)	individual level markov model
monte carlo model	monte carlo
queueing network model	queueing network
regression	regression
risk model	risk model
probability calculation	stochastic
convolution model	stochastic
stochastic model	stochastic
maximum likelihood model	stochastic
probability model	stochastic
lz model	stochastic
survival model	survival model
can*trol 2	unknown

5 Appendix H. Connection graphs

5.1 Model by Cancer

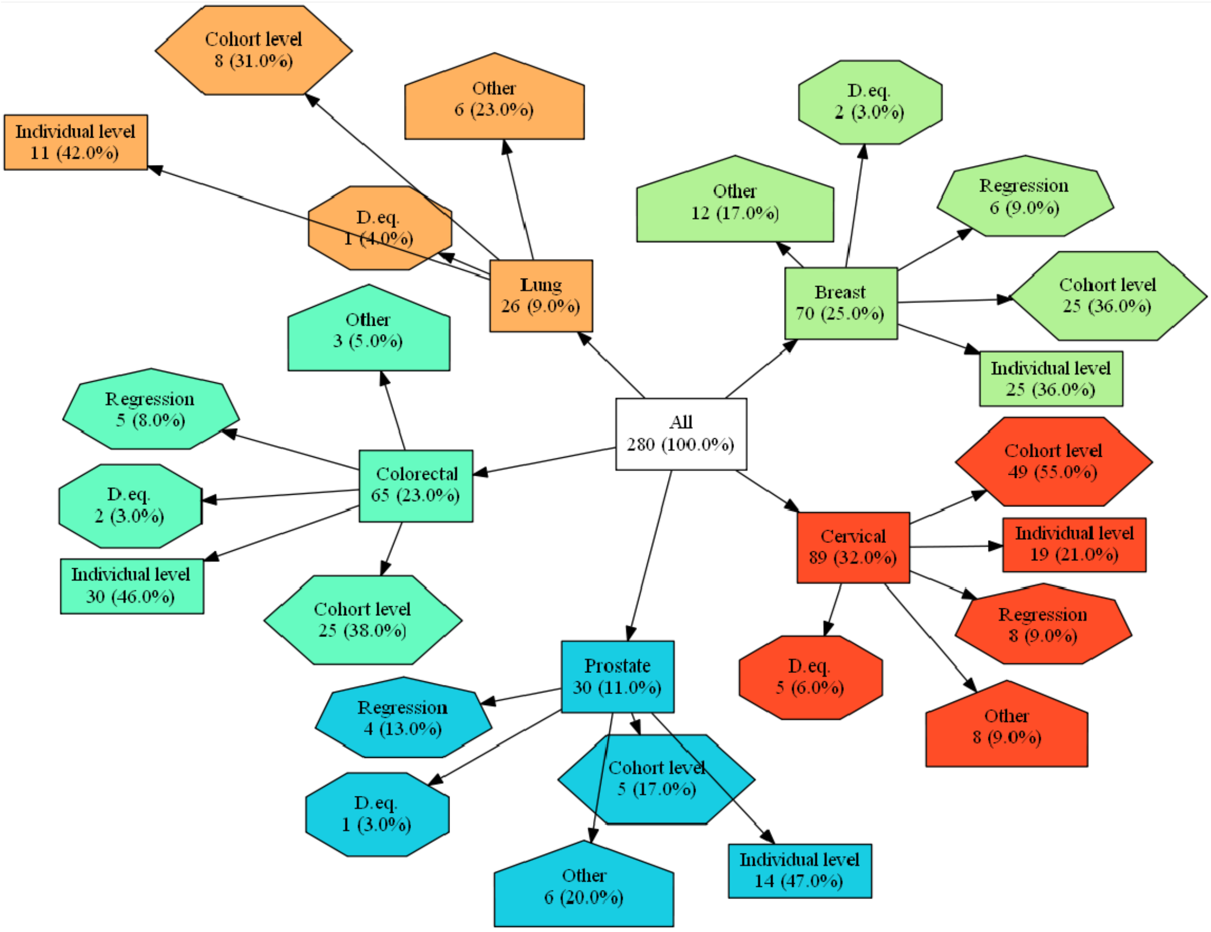

Figure 1: Number of studies in different world parts stratified by world cancer.

5.2 Cancer by population world part

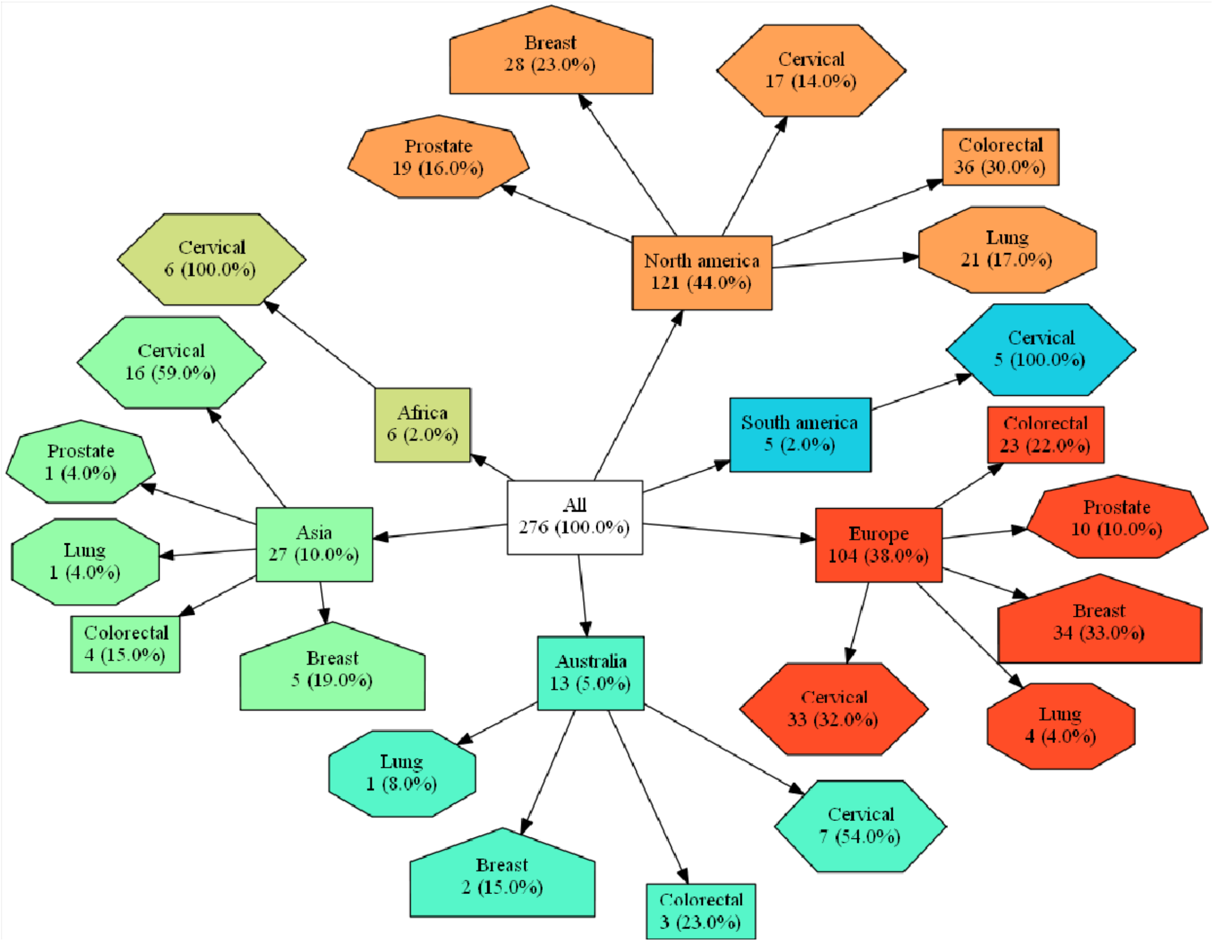

Figure 2: Number of studies in different world parts stratified by world parts.

5.3 Studies population geography by year

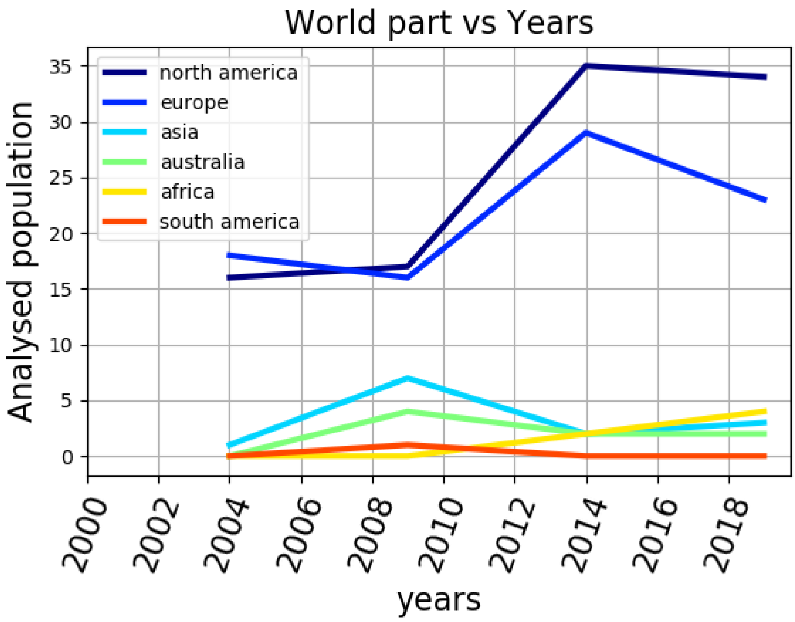

Figure 3: Studies population dynamics. Every point is the number of papers published during previous 5 years.

5.4 Studies publications dynamics

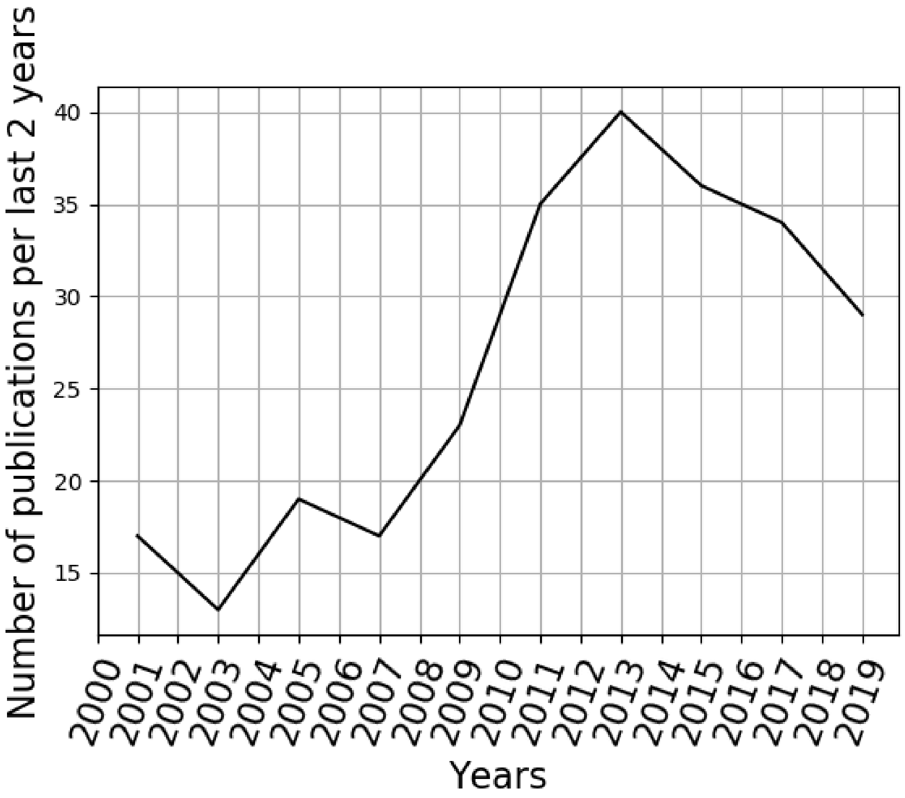

Figure 4: Studies publication dynamics. Every point is the number of papers published during previous 2 years.

6 Appendix I. List of chosen and analyzed publications

Authors	Name	Year
Louiza S Velentzis, Michael Caruana, Kate T Simms, Jie-Bin Lew, Ju-Fang Shi, Marion Saville, Megan A Smith, Sarah J Lord, Jeffrey Tan, Deborah Bateson, Michael Quinn, Karen Canfell	How Will Transitioning From Cytology To Hpv Testing Change The Balance Between The Benefits And Harms Of Cervical Cancer Screening? Estimates Of The Impact On Cervical Cancer, Treatment Rates And Adverse Obstetric Outcomes In Australia, A High Vaccination Coverage Country	2017
Gerrit Draisma, Joost Van Rosmalena	A Note On The Catch-Up Time Method For Estimating Lead Or Sojourn Time In Prostate Cancer Screening	2013
Rutter Carolyn M, Kim Jane J, Meester Reinier G S, Sprague Brian L, Burger Emily A, Zauber Ann G, Ergun Mehmet Ali, Campos Nicole G, Doubeni Chyke A, Trentham-Dietz Amy, Sy Stephen, Alagoz Oguzhan, Stout Natasha, Lansdorp-Vogelaar Iris, Corley Douglas A, Tosteson Anna N A	Effect Of Time To Diagnostic Testing For Breast Cervical And Colorectal Cancer Screening Abnormalities On Screening Efficacy A Modeling Study	2017
Armaroli P, Gallo F, Bellomi A, Ciatto S, Consonni D, Davi D, Giorgi-Rossi P, Iossa A, Mancini E, Naldoni C, Polla E, Ronco G, Serafini M, Vergini V, Zanier L, Zappa M, Segnan N	Do Women ¿50 Years Of Age Need As Much Screening As Women ¡50 Years After They Have Had Negative Screening Results	2008
Linda Sharp, Lesley Tilson, Sophie Whyte, Alan O Ceilleachair, Cathal Walsh, Cara Usher, Paul Tappenden,James Chilcott, Anthony Staines, Michael Barry, Harry Comber	Using Resource Modelling To Inform Decision Making And Service Planning: The Case Of Colorectal Cancer Screening In Ireland	2013
Cathy J Bradley, Iris Lansdorp-Vogelaar, K Robin Yabroff,Bassam Dahman, Angela Mariotto, Eric J Feuer, Martin L Brown,	Productivity Savings From Colorectal Cancer Prevention And Control Strategies	2011
Jeroen J Van Den Broek, Nicolien T Van Ravesteyn, Jeanne S Mandelblatt,Hui Huang,Mehmet Ali Ergun, Elizabeth S Burnside, Cong Xu, Yisheng Li,Oguzhan Alagoz, Sandra J Lee, Natasha K Stout, Juhee Song, Amy Trentham-Dietz,Sylvia K Plevritis, Sue M Moss, Harry J De Koning	Comparing Cisnet Breast Cancer Incidence And Mortality Predictions To Observed Clinical Trial Results Of Mammography Screening From Ages 40 To 49	2017
Gerrit Draisma, Renske Postma, Fritz H Schroder, Theo H Van Der Kwast, Harry J De Koning	Gleason Score Age And Screening Modeling Dedifferentiation In Prostate Cancer	2006
K Kerlikowske, P Salzmann, Ka Phillips, Ja Cauley, Sr Cummings	Continuing Screening Mammography In Women Aged 70 To 79 Years: Impact On Life Expectancy And Cost-Effectiveness.	1999
Jeff Ching-Fu Hsieh, Susanna M Cramb, James M Mcgree,Peter D Baade, Nathan Am Dunn, Kerrie L Mengersen	Bayesian Spatial Analysis For The Evaluation Of Breast Cancer Detection Methods	2014
Van Luijt Pa, Heijnsdijk Ea, Van Ravesteyn Nt, Hofvind S, De Koning Hj	Breast Cancer Incidence Trends In Norway And Estimates Of Overdiagnosis	2016
Ming-Shien Yen, Shan-Lin You, Nicole Ferko, Donna Debicki, Yi-Chen Chen, Cheng-Yang Chou	Estimating The Long-Term Clinical Impact Of Cervical Cancer Vaccination In Taiwan	2009
Wever Em, Draisma G, Heijnsdijk Ea, De Koning Hj	How Does Early Detection By Screening Affect Disease Progression Modeling Estimated Benefits In Prostate Cancer Screening	2011
Elissa M Ozanne, Laura J Esserman	Evaluation Of Breast Cancer Risk Assessment Techniques A Cost-Effectiveness Analysis	2004
Wong Io, Kuntz Km, Cowling Bj, Lam Cl, Leung Gm	Cost-Effectiveness Of Mammography Screening For Chinese Women	2007
Lee Th, Kim W, Shin J, Park Ec, Park S, Kim Th,	Strategic Distributional Cost-Effectiveness Analysis For Improving National Cancer Screening Uptake In Cervical Cancer A Focus On Regional Inequality In South Korea	2018

Matthias Arnold, Anne S Quante	Personalized Mammography Screening And Screening Adherence-A Simulation And Economic Evaluation	2018
Marshall D, Simpson Kn, Earle Cc, Chu C	Potential Cost-Effectiveness Of One-Time Screening For Lung Cancer (Lc) In A High Risk Cohort	2001
Wu D, Kafadar K, Rosner Gl, Broemeling Ld	The Lead Time Distribution When Lifetime Is Subject To Competing Risks In Cancer Screening	2012
Meng-Kan Chen, Hui-Fang Hung, Stephen Duffy, Amy Ming-Fang Yen, Hsiu-Hsi Chen	Cost-Effectiveness Analysis For Pap Smear Screening And Human Papillomavirus Dna Testing And Vaccination	2011
Karnon Jonathan, Jones Roy, Czoski-Murray Carolyn, Srnith Kevin J	Cost-Utility Analysis Of Screening High-Risk Groups For Anal Cancer	2008
Canfell K, Shi Jf, Lew Jb, Walker R, Zhao Fh, Simonella L, Chen Jf, Legood R, Smith, Nickson C, Qiao Yl	Prevention Of Cervical Cancer In Rural China Evaluation Of Hpv Vaccination And Primary Hpv Screening Strategies	2011
Sherry Yueh-Hsia Chiu, Nea Malila, Amy Ming-Fang Yen,Sam Li-Sheng Chen, Jean Ching-Yuan Fann, Matti Hakama	Predicting The Effectiveness Of The Finnish Population-Based Colorectal Cancer Screening Programme	2017
Reka Pataky, Roman Gulati, Ruth Etzioni, Peter Black, Kim N Chi, Andrew J Coldman,Tom Pickles, Scott Tyldesley, Stuart Peacock,	Is Prostate Cancer Screening Cost-Effective A Microsimulation Model Of Prostate-Specific Antigen-Based Screening For British Columbia Canada	2014
Uhry Z, Hdelin G, Colonna M, Asselain B, Arveux P, Exbrayat C, Guldenfelds C, Soler-Michel P, Molini F, Trtarre B, Rogel A, Courtial I, Danzon A, Guizard Av, Ancelle-Park R, Eilstein D, Duffy S	Modelling The Effect Of Breast Cancer Screening On Related Mortality Using French Data	2011
Lefevere E, Hens N, Theeten H, Van Den Bosch K, Beutels P, De Smet F, Van Damme P	Like Mother Like Daughter Mothers History Of Cervical Cancer Screening And Daughters Human Papillomavirus Vaccine Uptake In Flanders Belgium	2011
Chauvin P, Josselin Jm, Heresbach D	The Influence Of Waiting Times On Cost-Effectiveness: A Case Study Of Colorectal Cancer Mass Screening	2013
Summer S Han, Kevin Ten Haaf, William D Hazelton, Vidit N Munshi, Jihyoun Jeon,Saadet A Erdogan, Colden Johanson, Pamela M Mcmahon, Rafael Meza, Chung Yinkong, Eric J Feuer, Harry J De Koning, Sylvia K Plevritis	The Impact Of Overdiagnosis On The Selection Of Efficient Lung Cancer Screening Strategies	2017
Lejeune C, Arveux P, Dancourt V, Fagnani F, Bonithon-Kopp C, Faivre J	A Simulation Model For Evaluating The Medical And Economic Outcomes Of Screening Strategies For Colorectal Cancer	2003
Tiago M De Carvalho, Eveline Am Heijnsdijk, Harry J De Koning	Screening For Prostate Cancer In The Us? Reduce The Harms And Keep The Benefit	2014
J Michaelson, E Halpern, Db Kopans	Breast Cancer: Computer Simulation Method For Estimating Optimal Intervals For Screening.	1999
Adrian H Zai, Seokjin Kim, Arnold Kamis, Ken Hung, Jeremiah G Ronquillo,Henry C Chueh, Steven J Atlas,	Applying Operations Research To Optimize A Novel Population Management System For Cancer Screening	2013
Rob Boer, Harry De Koning, Anthony Threlfall, Peter Warmerdam, Andrew Street, Ellis Friedman,Ciaran Woodman	Cost-Effectiveness Of Shortening Screening Interval Or Extending Age Range Of Nhs Breast Screening Programme Computer Simulation Study	1998
Weedon-Fekjaer H, Tretli S, Aalen Oo	Estimating Screening Test Sensitivity And Tumour Progression Using Tumour Size And Time Since Previous Screening	2010
Hui-Chu Lang, Koyin Chang, Yung-Hsiang Ying	Quality Of Life Treatments And Patients Willingness To Pay For A Complete Remission Of Cervical Cancer In Taiwan	2011
F. J. Montz, Fredric L. Farber, Robert E. Bristow, Terri Cornelison	Impact Of Increasing Papanicolaou Test Sensitivity And Compliance A Modeled Cost And Outcomes Analysis	2001
Accetta G, Biggeri A, Carreras G, Lippi G, Carozzi Fm, Confortini M, Zappa M, Paci E	Is Human Papillomavirus Screening Preferable To Current Policies In Vaccinated And Unvaccinated Women A Cost-Effectiveness Analysis	2010

Iris Lansdorp-Vogelaar, Marjolein Van Ballegooijen, Ann G Zauber, Rob Boer, Janneke Wilschut, J Dik F Habbema	At What Costs Will Screening With Ct Colonography Be Competitive A Cost-Effectiveness Approach	2009
Van Ravesteyn Nt, Miglioretti Dl, Stout Nk, Lee Sj, Schechter Cb, Buist Ds, Huang H, Heijnsdijk Ea, Trentham-Dietz A, Alagoz O, Near Am, Kerlikowske K, Nelson Hd, Mandelblatt Js, De Koning Hj	Tipping The Balance Of Benefits And Harms To Favor Screening Mammography Starting At Age 40 Years A Comparative Modeling Study Of Risk	2012
Roman Gulati, Angela B Mariotto, Shu Chen, John L Gore, Ruth Etzioni	Long-Term Projections Of The Number Needed To Screen And Additional Number Needed To Treat In Prostate Cancer Screening	2011
Roman Gulati, Lurdes Y T Inoue, John L Gore, Jeffrey Katcher, Ruth Etzioni	Individualized Estimates Of Overdiagnosis In Screen-Detected Prostate Cancer	2014
Fisher R, Rooney Ps	Faecal Occult Blood Testing In Areas Of High Incidence And Mortality The Effect Of Screening The Population Aged 50 To 59	2010
Arnaud Seigneurin, Jose Labarere, Olivier Franois, Catherine Exbrayat,Maylis Dupouy, Marc Filippi, Marc Colonna	Overdiagnosis And Overtreatment Associated With Breast Cancer Mammography Screening A Simulation Study With Calibration To Population-Based Data	2016
Stone Ca, Carter Rc, Vos T, John Js	Colorectal Cancer Screening In Australia An Economic Evaluation Of A Potential Biennial Screening Program Using Faecal Occult Blood Tests	2004
Melnikow J, Tancredi Dj, Yang Z, Ritley D, Jiang Y, Slee C, Popova S, Rylett P, Knutson K, Smalley S	Program-Specific Cost-Effectiveness Analysis Breast Cancer Screening Policies For A Safety-Net Program	2013
Carolyn M Rutter, Amy B Knudsen, Tracey L Marsh,V Paul Doria-Rose, Eric Johnson, Chester Pabiniak,Karen M Kuntz, Marjolein Van Ballegooijen,Ann G Zauber, Iris Lansdorp-Vogelaar	Validation Of Models Used To Inform Colorectal Cancer Screening Guidelines Accuracy And Implications	2016
Anderson R, Haas M, Shanahan M	The Cost-Effectiveness Of Cervical Screening In Australia What Is The Impact Of Screening At Different Intervals Or Over A Different Age Range	2008
Gerrit Draisma , Ruth Etzioni , Alex Tsodikov , Angela Mariotto , Elisabeth Wever , Roman Gulati , Eric Feuer ,Harry De Koning	Lead Time And Overdiagnosis In Prostate-Specific Antigen Screening Importance Of Methods And Context	2009
Nicole G Campos, Vivien Tsu, Jose Jeronimo, Denise Njama-Meya, Mercy Mvundura And Jane J Kim	Cost-Effectiveness Of An Hpv Self-Collection Campaign In Uganda Comparing Models For Delivery Of Cervical Cancer Screening In A Low-Income Setting	2016
Hsin-Ju Hsieh, Tony Hsiu-Hsi Chen, Shu-Hui Chang	Assessing Chronic Disease Progression Using Non-Homogeneous Exponential Regression Markov Models An Illustration Using A Selective Breast Cancer Screening In Taiwan	2002
Eric J Feuer, Angela Mariotto, Ray Merrill	Modeling The Impact Of The Decline In Distant Stage Disease On Prostate Carcinoma Mortality Rates	2002
Gerrit Draisma, Rob Boer, Suzie J Otto, Ingrid W Van Der Cruijsen,Ronald A M Damhuis, Fritz H Schroder, Harry J De Koning	Lead Times And Overdetection Due To Prostate-Specific Antigen Screening Estimates From The European Randomized Study Of Screening For Prostate Cancer	2003
M-F Yena, L Tabar, B Vitak, Ra Smithe, H-H Chenf, Sw Duffy	Quantifying The Potential Problem Of Overdiagnosis Of Ductal Carcinoma In Situ In Breast Cancer Screening	2003
Sue J Goldie, Michele Kohli, Daniel Grima, Milton C Weinstein,Thomas C Wright, F Xavier Bosch, Eduardo Franco	Projected Clinical Benefits And Cost-Effectiveness Of A Human Papillomavirus 1618 Vaccine	2004
P F Pinsky	An Early- And Late-Stage Convolution Model For Disease Natural History	2004
Alexandra Barratt, Kirsten Howard, Les Irwig, Glenn Salkeld, Nehmat Houssami	Model Of Outcomes Of Screening Mammography Information To Support Informed Choices	2005

Renee Manser, Andrew Dalton, Rob Carter, Graham Byrnes,Mark Elwood, Donald A Campbell	Cost-Effectiveness Analysis Of Screening For Lung Cancer With Low Dose Spiral Ct Computed Tomography In The Australian Setting	2005
Ulrike Haug, Hermann Brenner	A Simulation Model For Colorectal Cancer Screening Potential Of Stool Tests With Various Performance Characteristics Compared With Screening Colonoscopy	2005
Uwe Siebert, Gaby Sroczynski, Peter Hillemanns, Jutta Engel,Roland Stabenow, Christa Stegmaier, Kerstin Voigt,Bernhard Gibis, Dieter Ho Lzel, Sue J Goldie	The German Cervical Cancer Screening Model Development And Validation Of A Decision-Analytic Model For Cervical Cancer Screening In Germany	2005
J H Groenewoud, J D M Otten, J Fracheboud, G Draisma, B M Van Ineveld, R Holland, A L M Verbeek, H J De Koning	Cost-Effectiveness Of Different Reading And Referral Strategies In Mammography Screening In The Netherlands	2006
Sylvia K Plevritis, Peter Salzman, Bronislava M Sigal, Peter W Glynn	A Natural History Model Of Stage Progression Applied To Breast Cancer	2006
Natasha K Stout, Marjorie A Rosenberg, Amy Trentham-Dietz, Maureen A Smith,Stephen M Robinson, Dennis G Fryback	Retrospective Cost-Effectiveness Analysis Of Screening Mammography	2006
Harald Weedon-Fekjr, Bo H Lindqvist, Lars J Vatten, Odd O Aalen, Steinar Tretli,	Breast Cancer Tumor Growth Estimated Through Mammography Screening Data	2008
C Bergeron, N Largeron, R Mcallister, P Mathevet, V Remy	Cost-Effectiveness Analysis Of The Introduction Of A Quadrivalent Human Papillomavirus Vaccine In France	2008
Pamela M Mcmahon,Chung Yin Kong, Milton C Weinstein, Angela C Tramontano, Lauren E Cipriano, Bruce E Johnson, Jane C Weeks, G Scott Gazelle	Adopting Helical Ct Screening For Lung Cancer Potential Health Consequences During A 15-Year Period	2008
Luz Myriam Reynales-Shigematsu, Eliane R Rodrigues, Eduardo Lazcano-Ponce	Cost-Effectiveness Analysis Of A Quadrivalent Human Papilloma Virus Vaccine In Mexico	2009
Andrea M Anonychuk, Chris T Bauch, Maraki Fikre Merid, Georges Van Kriekinge, Nadia Demarteau	A Cost-Utility Analysis Of Cervical Cancer Vaccination In Preadolescent Canadian Females	2009
Arthi Vijayaraghavan, Molly Efrusy, Gerhard Lindeque, Greta Dreyer, Christopher Santas	Cost-Effectiveness Of High-Risk Hpv Dna Testing For Cervical Cancer Screening In South Africa	2009
Deborah L Goldwasser,Marek Kimmel,	Modeling Excess Lung Cancer Risk Among Screened Arm Participants In The Mayo Lung Project	2009
Bjorn Berg, Brian Denton,Heidi Nelson,Hari Balasubramanian,Ahmed Rahman, Angela Bailey, Keith Lindor	A Discrete Event Simulation Model To Evaluate Operational Performance Of A Colonoscopy Suite	2009
Elisabeth M Wever, Gerrit Draisma, Eveline A M Heijnsdijk, Monique J Roobol, Rob Boer, Suzie J Otto, Harry J De Koning	Prostate-Specific Antigen Screening In The United States Vs In The European Randomized Study Of Screening For Prostate Cancer-Rotterdam	2010
Johannes Berkhof, Veerle M Coupe, Johannes A Bogaards, Folkert J Van Kemenade,Theo J Helmerhorst, Peter J Snijders, Chris J Meijer	The Health And Economic Effects Of Hpv Dna Screening In The Netherlands	2010
Mark Jit, Nadia Demarteau, Elamin Elbasha, Gary Ginsberg, Jane Kim, Naiyana Praditsitthikorn,Edina Sinanovic, Raymond Hutubessy	Human Papillomavirus Vaccine Introduction In Low-Income And Middle-Income Countries Guidance On The Use Of Cost-Effectiveness Models	2011
Brenda K Edwards,Elizabeth Ward,Betsy A Kohler, Christie Eheman, Ann G Zauber,Robert N Anderson,Ahmedin Jemal, Maria J Schymura,Iris Lansdorp-Vogelaar, Laura C Seeff, Marjolein Van Ballegooijen, S Luuk Goede, Lynn A G Ries	Annual Report To The Nation On The Status Of Cancer 1975-2006 Featuring Colorectal Cancer Trends And Impact Of Interventions Risk Factors Screening And Treatment To Reduce Future Rates	2009

Rafael Meza, Kevin Ten Haaf, Chung Yin Kong, Ayca Erdogan, William C Black, Martin C Tammemagi, Sung Eun Choi, Jihyoun Jeon, Summer S Han, Vidit Munshi,Joost Van Rosmalen, Paul Pinsky, Pamela M Mcmahon, Harry J De Koning, Eric J Feuer, William D Hazelton, Sylvia K Plevritis	Comparative Analysis Of 5 Lung Cancer Natural History And Screening Models That Reproduce Outcomes Of The Nlst And Plco Trials	2014
Uri Ladabaum, Angel Ferrandez, Angel Lanas	Cost-Effectiveness Of Colorectal Cancer Screening In High-Risk Spanish Patients Use Of A Validated Model To Inform Public Policy	2010
N B Gunsoy, M Garcia-Closas, S M Moss	Estimating Breast Cancer Mortality Reduction And Overdiagnosis Due To Screening For Different Strategies In The United Kingdom	2014
Tjalke A Westra, Irina Stirbu-Wagner, Sara Dorsman, Eric D Tutuhatunewa, Edwin L De Vrij, Hans W Nijman,Toos Daemen, Jan C Wilschut, Maarten J Postma	Inclusion Of The Benefits Of Enhanced Cross Protection Against Cervical Cancer And Prevention Of Genital Warts In The Cost-Effectiveness Analysis Of Human Papillomavirus Vaccination In The Netherlands	2013
Alex Van Der Steen, Amy B Knudsen, Frank Van Hees,Gailya P Walter, Franklin G Berger, Virginie G Daguise,Karen M Kuntz, Ann G Zauber, Marjolein Van Ballegooijen,Iris Lansdorp-Vogelaar	Optimal Colorectal Cancer Screening In States? Low-Income, Uninsured Populations The Case Of South Carolina	2015
T Kobayashi,R Goto, K Ito, K Mitsumori	Prostate Cancer Screening Strategies With Re-Screening Interval Determined By Individual Baseline Prostate-Specific Antigen Values Are Cost-Effective	2007
Inge Mcm De Kok, Ida J Korfage,Wilbert B Van Den Hout,Theo Jm Helmerhorst,J Dik F Habbema,Marie-Louise Essink-Bot,Marjolein Van Ballegooijen,	Quality Of Life Assumptions Determine Which Cervical Cancer Screening Strategies Are Cost-Effective	2018
Anneli Uuskla, Andres Mrsepp, Kosuke Kawai, Mait Raag, Mikk Jrisson, Matthew Pillsbury	The Epidemiological And Economic Impact Of Aqua-Drivalent Human Papillomavirus (Hpv) Vaccine In Estonia	2013
Natasha K Stout, Jeremy D Goldhaber-Fiebert, Jesse D Ortendahl, Sue J Goldie	Trade-Offs In Cervical Cancer Prevention Balancing Benefits And Risks	2008
Carolyn M Rutter, Jane J Kim, Reinier Gs Meester, Brian L Sprague,Emily A Burger, Ann G Zauber, Mehmet Ali Ergun, Nicole G Campos,Chyke A Doubeni, Amy Trentham-Dietz, Stephen Sy, Oguzhan Alagoz, Natasha Stout,Iris Lansdorp-Vogelaar, Douglas A Corley, Anna N A Tosteson	Effect Of Time To Diagnostic Testing For Breast, Cervical, And Colorectal Cancer Screening Abnormalities On Screening Efficacy: A Modeling Study	2017
Roman Gulati, Alex Tsodikov, Elisabeth M Wever, Angela B Mariotto, Eveline A M Heijnsdijk, Jeffrey Katcher, Harry J De Koning, Ruth Etzioni	The Impact Of Plco Control Arm Contamination On Perceived Psa Screening Efficacy	2012
F W Schultz, R Boer, H J De Koning	Chapter 7 Description Of Miscan-Lung The Erasmus Mc Lung Cancer Microsimulation Model For Evaluating Cancer Control Interventions	2012
Necdet B Gunsoy, Montserrat Garcia-Closas, Sue M Moss	Modelling The Overdiagnosis Of Breast Cancer Due To Mammography Screening In Women Aged 40to 49 In The United Kingdom	2012
J Luttjeboer, Ta Westra, Jc Wilschut, Hw Nijman, T Daemen, Mj Postma	Cost-Effectiveness Of The Prophylactic Hpv Vaccine An Application To The Netherlands Taking Non-Cervical Cancers And Cross-Protection Into Account	2013
Wilm Quentin, Yaw Adu-Sarkodie, Fern Terris-Prestholt, Rosa Legood, Baafuor K Opoku,Philippe Mayaud	Costs Of Cervical Cancer Screening And Treatment Using Visual Inspection With Acetic Acid Via And Cryotherapy In Ghana The Importance Of Scale	2011
Giedrius Vanagas, Zilvinas Padaiga	Healthcare Spending In The Case Of A Hpv1618 Population-Wide Vaccination Programme	2012

Christine L Barnett, Scott A Tomlins, Daniel J Underwood,John T Wei, Todd M Morgan, James E Montie, Brian T Denton	Two-Stage Biomarker Protocols For Improving The Precision Of Early Detection Of Prostate Cancer	2017
Hunter Dj, Drake Sm, Shortt Se, Dorland Jl, Tran N	Simulation Modeling Of Change To Breast Cancer Detection Age Eligibility Recommendations In Ontario 2002-2021	2004
Fs Mennini, P Giorgi Rossi, F Palazzo, N Largeron,	Health And Economic Impact Associated With A Quadrivalent Hpv Vaccine In Italy	2009
Gomez Ja, Lepetic A, Demarteau N	Health Economic Analysis Of Human Papillomavirus Vaccines In Women Of Chile Perspective Of The Health Care Payer Using A Markov Model	2014
By Joshua A Roth, Sean D Sullivan, Bernardo Hl Goulart,Arliene Ravelo, Joanna C Sanderson, Scott D Ramsey	Projected Clinical Resource Use And Fiscal Impacts Of Implementing Low-Dose Computed Tomography Lung Cancer Screening In Medicare	2015
Chen-Yang Hsu, Ming-Fang Yen, Anssi Auvinen, Yueh-Hsia Chiu, Hsiu-Hsi Chen	Bayesian Negative-Binomial-Family-Based Multistate Markov Model For The Evaluation Of Periodic Population-Based Cancer Screening Considering Incomplete Information And Measurement Errors	2016
Tay K, Tay Sk	The Impact Of Cytology Screening And Hpv Vaccination On The Burden Of Cervical Cancer	2011
Jeanne S Mandelblatt, Clyde B Schechter, K Robin Yabroff,William Lawrence, James Dignam, Martine Extermann,Sarah Fox, Gretchen Orosz, Rebecca Silliman,Jennifer Cullen, Lodovico Balducci	Toward Optimal Screening Strategies For Older Women Costs Benefits And Harms Of Breast Cancer Screening By Age Biology And Health Status	2005
Subramanian Sujha, Bobashev Georgiy, Morris Robert J, Hoover Sonja	Personalized Medicine For Prevention: Can Risk Stratified Screening Decrease Colorectal Cancer Mortality At An Acceptable Cost?	2017
Georg Ruile, Anatoli Djanatliev, Christine Kriza,Florian Meier, Ines Leb,Willi A Kalender, Peter L Kolominsky-Rabas	Screening For Breast Cancer With Breast-Ct In A Prohta Simulation	2015
Mvundura M, Tsu V	Estimating The Costs Of Cervical Cancer Screening In High-Burden Sub-Saharan African Countries	2014
Anton Palma, David Lounsbury, Nicolas Schlecht, Ilir Agalliu	A System Dynamics Model Of Serum Prostate-Specific Antigen Screening For Prostate Cancer	2015
Jos Luttjeboer, Didik Setiawan, Qi Cao, Toos Cahh Daemen, Maarten J Postma	Threshold Cost-Effectiveness Analysis For A Therapeutic Vaccine Against Hpv-1618-Positive Cervical Intraepithelial Neoplasia In The Netherlands	2016
Matthijsse Sm, Van Rosmalen J, Hontelez Ja, Bakker R, De Kok Im, Van Ballegooijen M, De Vlas Sj	The Role Of Acquired Immunity In The Spread Of Human Papillomavirus Hpv Explorations With A Microsimulation Model	2015
Lee Kj, Inoue M, Otani T, Iwasaki M, Sasazuki S, Tsugane S Japan Public Health Center-Based Prospective Study	Colorectal Cancer Screening Using Fecal Occult Blood Test And Subsequent Risk Of Colorectal Cancer A Prospective Cohort Study In Japan	2007
Murray D Krahn, Karen E Bremner, Brandon Zagorski,Shabbir M H Alibhai, Wendong Chen, George Tomlinson,Nicholas Mitsakakis, Gary Naglie	Health Care Costs For State Transition Models In Prostate Cancer	2013
Leslie Anne Campbell, John T Blake, George Kephart,Eva Grunfeld, Donald Macintosh,	Understanding The Effects Of Competition For Constrained Colonoscopy Services With The Introduction Of Population-Level Colorectal Cancer Screening A Discrete Event Simulation Model	2016
Sheehan Df, Criss Sd, Gazelle Gs, Pandharipande Pv, Kong Cy,	Evaluating Lung Cancer Screening In China Implications For Eligibility Criteria Design From A Microsimulation Modeling Approach	2017
Kim E Jeong, John A Cairns	Review Of Economic Evidence In The Prevention And Early Detection Of Colorectal Cancer	2013

Diego Munoz, Aimee M Near, Nicolien T Van Ravesteyn, Sandra J Lee, Clyde B Schechter, Oguzhan Alagoz,Donald A Berry, Elizabeth S Burnside, Yaojen Chang, Gary Chisholm, Harry J De Koning, Mehmet Ali Ergun,Eveline A M Heijnsdijk, Hui Huang, Natasha K Stout, Brian L Sprague, Amy Trentham-Dietz, Jeanne S Mandelblatt, Sylvia K Plevritis	Effects Of Screening And Systemic Adjuvant Therapy On Er-Specific Us Breast Cancer Mortality	2014
Ruth Etzioni, Alex Tsodikov, Angela Mariotto, Aniko Szabo, Seth Falcon, Jakewegelin, Dante Ditommaso, Kent Karnofski, Roman Gulati, David F Penson, Eric Feuer	Quantifying The Role Of Psa Screening In The Us Prostate Cancer Mortality Decline	2008
Zoltan Voko, Laszlo Nagyjanosi, Zoltan Kalo	Cost-Effectiveness Of Adding Vaccination With The As04-Adjuvanted Human Papillomavirus 16/18 Vaccine To Cervical Cancer Screening In Hungary	2012
Sharma M, Sy S, Kim Jj	The Value Of Male Human Papillomavirus Vaccination In Preventing Cervical Cancer And Genital Warts In A Low-Resource Setting	2015
Paul D P Pharoah, Bernadette Sewell, Deborah Fitzsimmons, Hayley S Bennett,Nora Pashayan	Cost-Effectiveness Of The Nhs Breast Screening Programme: Life Table Model	2013
Andrew M D Wolf, Elizabeth T H Fontham, Drph Timothy R Church, Christopher R Flowers, Carmen E Guerra, Samuel J Lamonte, Ruth Etzioni, Matthew T Mckenna, Kevin C Oeffinger, Ya-Chen Tina Shih, Louise C Walter, Kimberly S Andrews, Ba Otis W Brawley, Durado Brooks, Stacey A Fedewa, Deana Manassaram-Baptiste, Rebecca L Siegel, Richard C Wender, Robert A Smith	Colorectal Cancer Screening For Average-Risk Adults 2018 Guideline Update From The American Cancer Society	2018
C Sherlaw-Johnson, Z Philips	An Evaluation Of Liquid-Based Cytology And Human Papillomavirus Testing Within The Uk Cervical Cancer Screening Programme	2004
Frank Van Hees, J Dik F Habbema, Reinier G Meester, Iris Lansdorp-Vogelaar, Marjolein Van Ballegooijen, Ann G Zauber	Should Colorectal Cancer Screening Be Considered In Elderly Persons Without Previous Screening? A Cost-Effectiveness Analysis	2014
Prudence Creighton, Jie-Bin Lew, Mark Clements, Megan Smith, Kirsten Howard, Suzanne Dyer,Sarah Lord, Karen Canfell,	Cervical Cancer Screening In Australia: Modelled Evaluation Of The Impact Of Changing The Recommended Interval From Two To Three Years	2010
Pamela M Mcmahon, Rafael Meza, Sylvia K Plevritis, William C Black, C Martin Tammemagi,Ayca Erdogan, Kevin Ten Haaf, William Hazelton, Theodore R Holford, Jihyoun Jeon,Lauren Clarke, Chung Yin Kong, Sung Eun Choi, Vidit N Munshi, Summer S Han,Joost Van Rosmalen, Paul F Pinsky, Suresh Moolgavkar, Harry J De Koning, Eric J Feuer	Comparing Benefits From Many Possible Computed Tomography Lung Cancer Screening Programs Extrapolating From The National Lung Screening Trial Using Comparative Modeling	2014
R G Blanks, M Waller, A Sanchez-Galvez, S M Moss	Monitoring And Evaluating The Performance Of The Uk Nhs Cervical Screening Programme Monitoring Performance By Using Cytology Outcomes Adjusted For Population Characteristics	2004
Xiuting Mo, Ruoyan Gai Tobe, Lijie Wang, Xianchen Liu, Bin Wu, Huiwen Luo, Chie Nagata,Rintaro Mori, Takeo Nakayama	Cost-Effectiveness Analysis Of Different Types Of Human Papillomavirus Vaccination Combined With A Cervical Cancer Screening Program In Mainland China	2017
Rick J Jansen, Bruce H Alexander, Kristin E Anderson, Timothy R Church	Quantifying Lead-Time Bias In Risk Factor Studies Of Cancer Through Simulation	2013

Helen Beer, Sam Hibbitts, Sinead Brophy, Rahman,Jo Waller, Shantini Paranjothy,	Does The Hpv Vaccination Programme Have Implications For Cervical Screening Programmes In The Uk?	2014
Oscar Andres-Gamboa,Liliana Chicaza, Mario Garca-Molina,Jorge Daz, Mauricio Gonzlez, Ral Murillo,Mnica Ballesteros, Ricardo Snchez	Cost-Effectiveness Of Conventional Cytology And Hpv Dna Testing For Cervical Cancer Screening In Colombia	2008
Paul F Pinsky	Estimation And Prediction For Cancer Screening Models Using Deconvolution And Smoothing	2001
Yamamoto N, Mori R, Jacklin P, Osuga Y, Kawana K, Shibuya K, Taketani Y	Introducing Hpv Vaccine And Scaling Up Screening Procedures To Prevent Deaths From Cervical Cancer In Japan A Cost-Effectiveness Analysis	2011
Carmen Armero, Antonio Lopez-Quilez, Rut Lopez-Sanchez	Bayesian Assessment Of Times To Diagnosis In Breast Cancer Screening	2008
Lu Shi, Haijun Tian, William J Mccarthy, Barbara Berman, Shinyi Wu, Rob Boer	Exploring The Uncertainties Of Early Detection Results Model-Based Interpretation Of Mayo Lung Project	2011
Ruth Etzioni, David F Penson, Julie M Legler, Dante Di Tommaso, Rob Boer,Peter H Gann, Eric J Feuer	Overdiagnosis Due To Prostate-Specific Antigen Screening Lessons From Us Prostate Cancer Incidence Trends	2002
Ray S Lin, Sylvia K Plevritis	Comparing The Benefits Of Screening For Breast Cancer And Lung Cancer Using A Novel Natural History Model	2012
Roberts Stephen, Wang Lijun, Klein Robert, Ness Reid, Dittus Robert	Development Of A Simulation Model Of Colorectal Cancer	2007
Blanks Rg, Moss Sm, Denton K	Improving The Nhs Cervical Screening Laboratory Performance Indicators By Making Allowance For Population Age Risk And Screening Interval	2006
Sopina E, Ashton T	Cost-Effectiveness Of A Cervical Screening Program With Human Papillomavirus Vaccine	2011
Praditsitthikorn N, Teerawattananon Y, Tantivess S, Limwattananon S, Riewpaiboon A, Chichareon S, Ieumwananonthachai N, Tangcharoensathien V	Economic Evaluation Of Policy Options For Prevention And Control Of Cervical Cancer In Thailand	2011
Sonnenberg A, Delco F	Cost-Effectiveness Of A Single Colonoscopy In Screening For Colorectal Cancer	2002
Ks Ross, Hb Carter, Jd Pearson, Ha Guess	Comparative Efficiency Of Prostate-Specific Antigen Screening Strategies For Prostate Cancer Detection.	2000
Dewilde S, Anderson R	The Cost-Effectiveness Of Screening Programs Using Single And Multiple Birth Cohort Simulations A Comparison Using A Model Of Cervical Cancer	2004
Vanni T, Luz Pm, Foss A, Mesa-Frias M, Legood R	Economic Modelling Assessment Of The Hpv Quadrivalent Vaccine In Brazil A Dynamic Individual-Based Approach	2012
Tramontano Ac, Sheehan Df, Mcmahon Pm, Dowling Ec, Holford Tr, Ryczak K, Lesko Sm, Levy Dt, Kong Cy	Evaluating The Impacts Of Screening And Smoking Cessation Programmes On Lung Cancer In A High-Burden Region Of The Usa A Simulation Modelling Study	2016
Nicole G Campos, Monisha Sharma, Andrew Clark, Kyueun Lee, Fangli Geng, Catherine Regan, Jane Kim, Stephen Resch	The Health And Economic Impact Of Scaling Cervical Cancer Prevention In 50 Low- And Lower-Middle-Income Countries	2017
Timmers Jm, Verbeek Al, Inthout J, Pijnappel Rm, Broeders Mj, Den Heeten Gj	Breast Cancer Risk Prediction Model A Nomogram Based On Common Mammographic Screening Findings	2013
Iris Lansdorp-Vogelaar, Karen M Kuntz, Amy B Knudsen, Janneke A Wilschut, Ann G Zauber, Marjolein Van Ballegooijen,	Stool Dna Testing To Screen For Colorectal Cancer In The Medicare Population	2010
Philips, Z, Whynes, Dk	Early Withdrawal From Cervical Cancer Screening The Question Of Cost-Effectiveness	2001
Janes H, Pepe M, Kooperberg C, Newcomb P	Identifying Target Populations For Screening Or Not Screening Using Logic Regression	2005

Mandelblatt Js, Cronin Ka, Bailey S, Berry Da, De Koning Hj, Draisma G, Huang H, Lee Sj, Munsell M, Plevritis Sk, Ravdin P, Schechter Cb, Sigal B, Stoto, Stout Nk, Van Ravesteyn Nt, Venier J, Zelen M, Feuer Ej Breast Cancer Working Group Of The Cancer Intervention, Surveillance Modeling Network	Effects Of Mammography Screening Under Different Screening Schedules Model Estimates Of Potential Benefits And Harms	2009
Iol Wong, Km Kuntz, Bj Cowling, Clk Lam, Gm Leung	Cost-Effectiveness Analysis Of Mammography Screening In Hong Kong Chinese Using State-Transition Markov Modelling	2010
Seppnen J, Heinvaara S, Hakulinen T	Predicting Impacts Of Mass-Screening Policy Changes On Breast Cancer Mortality	2008
Harrell W Chesson, Donatus U Ekwueme, Mona Saraiya, Lauri E Markowitz	Cost-Effectiveness Of Human Papillomavirus Vaccination Inthe United States	2008
Miriam P Van Der Meulen, Atija Kapidzic, Monique E Van Leerdam,Alex Van Der Steen, Ernst J Kuipers, Manon Cw Spaander,Harry J De Koning, Lieke Hol, Iris Lansdorp-Vogelaar	Do Men And Women Need To Be Screened Differently With Fecal Immunochemical Testing A Cost-Effectiveness Analysis	2017
S W Duffy, J K Field, P C Allgood, A Seigneurin	Translation Of Research Results To Simple Estimates Of The Likely Effect Of A Lung Cancer Screening Programme In The United Kingdom	2014
T C Prevost, G Launoy, S W Duffy, H H Chen	Estimating Sensitivity And Sojourn Time In Screening For Colorectal Cancer A Comparison Of Statistical Approaches	1998
Rue M, Vilaprinyo E, Lee S, Martinez-Alonso M, Carles, Marcos-Gragera R, Pla R, Espinas Ja	Effectiveness Of Early Detection On Breast Cancer Mortality Reduction In Catalonia Spain	2009
Bending Mw, Trueman P, Lowson Kv, Pilgrim H, Tappenden P, Chilcott J, Tappenden J	Estimating The Direct Costs Of Bowel Cancer Services Provided By The National Health Service In England	2010
Elisabeth Fp Peterse, Reinier Gs Meester, Rebecca L Siegel, Jennifer C Chen, Andrea Dwyer, Dennis J Ahnen, Robert A Smith, Ann G Zauber, Iris Lansdorp-Vogelaar,	The Impact Of The Rising Colorectal Cancer Incidence In Young Adults On The Optimal Age To Start Screening Microsimulation Analysis I To Inform The American Cancer Society Colorectal Cancer Screening Guideline	2018
Kathryn Fitch, Pyenson Bruce, Blumen Helen, Weisman Thomas, Small Art	The Value Of Colonoscopic Colorectal Cancer Screening Of Adults Aged 50 To 64 Years	2015
F Loeve, R Boer, Gj Van Oortmarssen, M Van Ballegooijen, Jdf Habbema	Impact Of Systematic False-Negative Test Results On The Performance Of Faecal Occult Blood Screening	2001
Kate T Simms, Jean-Franois Laprise, Megan A Smith, Jie-Bin Lew, Michael Caruana, Marc Brisson, Karen Canfell	Cost-Effectiveness Of The Next Generation Nonavalent Human Papillomavirus Vaccine In The Context Of Primary Human Papillomavirus Screening In Australia A Comparative Modelling Analysis	2016
Marjolein Van Ballegooijen, Rob Boer, Ann G Zauber	Simulation Of Colorectal Cancer Screening: What We Do And Do Not Know And Does It Matter	2010
Johannes Berkhof, Martine C De Bruijne, Gilda D Zielinski, Nicole Wj Bulkmans, Lawrence Rozendaal,Peter Jf Snijders, Rene Hm Verheijen, Chris Jlm Meijer	Evaluation Of Cervical Screening Strategies With Adjunct High-Risk Human Papillomavirus Testing For Women With Borderline Or Mild Dyskaryosis	2006
Murray D Krahn, B Ann Coombs, Isra G Levy	Current And Projected Annual Direct Costs Of Screening Asymptomatic Men For Prostate Cancer Using Prostate-Specific Antigen	1999
Rm Rogoza, Tawestra, N Ferko, Jj Tamminga, Mf Drummond,T Daemen, Jc Wilschut, Mj Postma,	Cost-Effectiveness Of Prophylactic Vaccination Against Human Papillomavirus 1618 For The Prevention Of Cervical Cancer Adaptation Of An Existing Cohort Model To The Situation In The Netherlands	2009
Mandelblatt Js, Lawrence Wf, Womack Sm, Jacobson D, Yi B, Hwang Yt, Gold K, Barter J, Shah K	Benefits And Costs Of Using Hpv Testing To Screen For Cervical Cancer.	2002
M Ramos, S Ferrer, J I Villaescusa, G Verdu, M D Salas, M D Cuevas	Use Of Risk Projection Models To Estimate Mortality And Incidence From Radiation-Induced Breast Cancer In Screening Programs	2005

Nicole G Campos, Jose Jeronimo, Vivien Tsu, Philip E Castle, Mercy Mvundura, Jane J Kima	The Cost-Effectiveness Of Visual Triage Of Human Papillomavirus-Positive Women In Three Low- And Middle-Income Countries	2017
Kim Jj, Kobus Ke, Diaz M, Oshea M, Van Minh H, Goldie Sj	Exploring The Cost-Effectiveness Of Hpv Vaccination In Vietnam: Insights For Evidence-Based Cervical Cancer Prevention Policy	2008
Karl Ulrich Petry, Franziska Rinnau, Gerd Bhmer, Bettina Hollwitz, Alexander Luyten, Nina Buttmann,Martin Brnger, Thomas Iftner	Annual Papanicolaou Screening For 5 Years Among Human Papillomavirus-Negative Women	2013
Gocgun Y, Banjevic D, Taghipour S, Montgomery N, Harvey Bj, Jardine Ak, Miller Ab	Cost-Effectiveness Of Breast Cancer Screening Policies Using Simulation	2015
Berhane, K, Weissfeld, La	Inference In Spline-Based Models For Multiple Time-To-Event Data, With Applications To A Breast Cancer Prevention Trial	2003
Duffy Sw, Raji Oy, Agbaje Of, Allgood Pc, Cassidy A, Field Jk	Use Of Lung Cancer Risk Models In Planning Research And Service Programs In Ct Screening For Lung Cancer	2009
Ea Burger, Jd Ortendahl, S Sy, Is Kristiansen, Jj Kim	Cost-Effectiveness Of Cervical Cancer Screening With Primary Human Papillomavirus Testing In Norway	2012
Iris Lansdorp-Vogelaar, Karen M Kuntz, Amy B Knudsen, Marjolein Van Ballegooijen,Ann G Zauber, Ahmedin Jemal	Contribution Of Screening And Survival Differences To Racial Disparities In Colorectal Cancer Rates	2012
Van Ballegooijen M, Van Den Akker-Van Marle E, Patnick J, Lynge E, Arbyn M, Anttila A, Ronco G, Dik J, Habbema F	Overview Of Important Cervical Cancer Screening Process Values Ineuropean Union (Eu) Countries, And Tentative Predictions Of The Corresponding Effectiveness And Cost-Effectiveness	2000
Rafia R, Brennan A, Madan J, Collins K, Reed Mw, Lawrence G, Robinson T, Greenberg D, Wyld L	Modeling The Cost-Effectiveness Of Alternative Upper Age Limits For Breast Cancer Screening In England And Wales	2015
Boyka Stoykova,Georgi Kuzmanov,Robin Dowie	Putting National Institute For Health And Clinical Excellence Guidance Into Practice A Cost Minimization Model Of A National Roll-Out Of Liquid Based Cytology In England	2008
Ekwueme Du, Uzunangelov Vj, Hoerger Tj, Miller Jw, Saraiya M, Benard Vb, Hall Ij, Royalty J, Li C, Myers Er	Impact Of The National Breast And Cervical Cancer Early Detection Program On Cervical Cancer Mortality Among Uninsured Low-Income Womenin The U.S., 1991?2007	2014
Li-Sheng Chen, Chao-Sheng Liao, Shu-Hui Chang, Hsin-Chih Lai, Tony Hsiu-Hsi Chen	Cost-Effectiveness Analysis For Determining Optimal Cut-Off Of Immunochemical Faecal Occult Blood Test For Population-Based Colorectal Cancer Screening (Kcis 16)	2007
Konno R, Sasagawa T, Fukuda T, Van Kriekinge G, Demarteau N	Cost-Effectiveness Analysis Of Prophylactic Cervical Cancer Vaccination In Japanese Women	2010
Shalini L Kulasingam, Raghu Rajan, Yvan St Pierre, C Victoria Atwood, Evan R Myers, Eduardo L Franco	Human Papillomavirus Testing With Pap Triage For Cervical Cancer Prevention In Canada: A Cost-Effectiveness Analysis	2009
Cronin Paula, Goodall Stephen, Lockett Trevor, Okeefe Christine, Norman Richard, Church Jody	Cost-Effectiveness Of An Advance Notification Letter To Increase Colorectal Cancer Screening	2013
Seigneurin A, Labarre J, Duffy Sw, Colonna M	Overdiagnosis Associated With Breast Cancer Screening A Simulation Study To Compare Lead-Time Adjustment Methods	2015
K K F Tsoi, S S M Ng, M C M Leung, J J Y Sung	Cost-Effectiveness Analysis On Screening For Colorectal Neoplasm And Management Of Colorectal Cancer In Asia	2008
Laura A Mclay, Christodoulos Foufoulides, Jason R W Merrick	Using Simulation-Optimization To Construct Screening Strategies For Cervical Cancer	2010
Jaakko Nevalainen, Ulf-Hkan Stenman, Teuvo L Tammela, Monique Roobol,Sigrid Carlsson, Kirsi Talala, Fritz H Schrder, Anssi Auvinen	What Explains The Differences Between Centres In The European Screening Trial A Simulation Study	2017
Canfell K, Barnabas R, Patnick J, Beral V	The Predicted Effect Of Changes In Cervical Screening Practice In The Uk Results From A Modelling Study	2004
Stuart G Baker	Evaluating The Age To Begin Periodic Breast Cancer Screening Using Data From A Few Regularly Scheduled Screenings	1998

Ruth Etzioni, Roman Gulati, Seth Falcon, David F Penson	Impact Of Psa Screening On The Incidence Of Advanced Stage Prostate Cancer In The United States A Surveillance Modeling Approach	2008
Saini Sd, Schoenfeld P, Vijan S	Can The Adenoma Detection Rate Reliably Identify Low-Performing Endoscopists Results Of A Modeling Study	2013
Coldman A J, Phillips N, Brisson J, Flanagan W, Wolfson M, Nadeau C, Fitzgerald N, Miller A B	Using The Cancer Risk Management Model To Evaluate Colorectal Cancer Screening Options For Canada	2013
Johannes Berkhof, Johannes A Bogaards, Erhan Demirel, Mireia Diaz,Monisha Sharma, Jane J Kim	Cost-Effectiveness Of Cervical Cancer Prevention In Central And Eastern Europe And Central Asia	2013
Loeve F, Boer R, Van Oortmarssen Gj, Van Ballegooijen M, Habbema Jdf	The Miscan-Colon Simulation Model For The Evaluation Of Colorectal Cancer Screening	1999
Akhila Balasubramanian, Shalini L Kulasingam, Atar Baer,James P Hughes, Evan R Myers, Constance Mao,Nancy B Kiviat, Laura A Koutsky	Accuracy And Cost-Effectiveness Of Cervical Cancer Screening By High-Risk Human Papillomavirus Dna Testing Of Self-Collected Vaginal Samples	2010
Sandra J Lee, Xiaoxue Li, Hui Huang, Marvin Zelen	The Dana-Farber Cisnet Model For Breastcancer Screening Strategies: An Update	2017
Reinier G S Meester, Elisabeth F P Peterse, Amy B Knudsen, Anne C De Weerdt, Jennifer C Chen, Anna P Lietz, Ba Andrea Dwyer, Dennis J Ahnen, Rebecca L Siegel, Robert A Smith, Ann G Zauber, Iris Lansdorp-Vogelaar	Optimizing Colorectal Cancer Screening By Race And Sex Microsimulation Analysis Ii To Inform The American Cancer Society Colorectal Cancer Screening Guideline	2018
Jenny Chia-Yun Wu, Matti Hakama, Ahti Anttila, Amy Ming-Fang Yen, Nea Malila, Tytti Sarkeala, Anssi Auvinen, Sherry Yueh-Hsia Chiu, Hsiu-Hsi Chen	Estimation Of Natural History Parameters Of Breast Cancer Based On Non-Randomized Organized Screening Data Subsidiary Analysis Of Effects Of Inter-Screening Interval Sensitivity And Attendance Rate On Reduction Of Advanced Cancer	2010
Howard K, Salkeld G, Irwig L, Adelstein Ba	High Participation Rates Are Not Necessary For Cost-Effective Colorectal Cancer Screening	2005
Amy Trentham-Dietz, Mehmet Ali Ergun, Oguzhan Alagoz, Natasha K Stout, Ronald E Gangnon, John M Hampton, Kim Dittus, Ted A James, Pamela M Vacek, Sally D Herschorn, Elizabeth S Burnside, Anna N A Tosteson, Donald L Weaver, Brian L Sprague	Comparative Effectiveness Of Incorporating A Hypothetical Dcis Prognostic Marker Into Breast Cancer Screening	2017
Jj Kim, Gm Leung, Pps Woo, Sj Goldie	Cost-Effectiveness Of Organized Versus Opportunistic Cervical Cytology Screening In Hong Kong	2004
Chauvin P, Josselin Jm, Heresbach D	Incremental Net Benefit And Acceptability Of Alternative Health Policies A Case Study Of Mass Screening For Colorectal Cancer	2011
Haghighat Shahpar, Akbari Mohammad Esmaeil, Yavari Parvin, Javanbakht Mehdi, Ghaffari Shahram	Cost-Effectiveness Of Three Rounds Of Mammography Breast Cancer Screening In Iranian Women	2016
David K Whynes, Aileen R Neilson, Andrew R Walker, Jack D Hardcastle	Faecal Occult Blood Screening For Colorectal Cancer Is It Cost-Effective	1998
Rebecca Landy, Peter Windridge, Matthew S Gillman, Peter D Sasieni	What Cervical Screening Is Appropriate For Women Who Have Been Vaccinated Against High Risk Hpv? A Simulation Study	2018
Alex Van Der Steen, Amy B Knudsen, Frank Van Hees,Gailya P Walter, Franklin G Berger, Virginie G Daguise,Karen M Kuntz, Ann G Zauber, Marjolein Van Ballegooijen,Iris Lansdorp-Vogelaar	Optimal Colorectal Cancer Screening In States? Low-Income, Uninsured Populations The Case Of South Carolina	2015
Wang Pe, Wang Tt, Chiu Yh, Yen Am, Chen Th	Evolution Of Multiple Disease Screening In Keelung A Model For Community Involvement In Health Interventions	2006

Rezaul K Khandker,Jane D Dulski,Jeffrey B Kilpatrick,Randall P Ellis,Janet B Mitchell, William B Baine	A Decision Model And Cost-Effectiveness Analysis Of Colorectal Cancer Screening And Surveillance Guidelines For Average-Risk Adults	2000
Nahvijou A, Daroudi R, Tahmasebi M, Amouzegar Hashemi F, Rezaei Hemami M, Akbari Sari A, Barati Marenani A, Zendehdel K	Cost-Effectiveness Of Different Cervical Screening Strategies In Islamic Republic Of Iran A Middle-Income Country With A Low Incidence Rate Of Cervical Cancer	2016
Kim S, Wu D	Estimation Of Sensitivity Depending On Sojourn Time And Time Spent In Preclinical State	2014
Irmgard Schiller-Fruehwirth, Beate Jahn, Patrick Einzinger,Gunther Zauner,Christoph Urach, Uwe Siebert	The Long-Term Effectiveness And Cost-Effectiveness Of Organized Versus Opportunistic Screening For Breast Cancer In Austria	2017
Amy B Knudsen, Chin Hur, G Scott Gazelle, Deborah Schrag, Elizabeth G Mcfarland, Karen M Kuntz	Rescreening Of Persons With A Negative Colonoscopy Result Results From A Microsimulation Model	2012
Beemsterboer, Pmm, Warmerdam, Pg, Boer, R, Borras, Jm, Moreno, V, Viladiu, P, De Koning, Hj	Screening For Breast Cancer In Catalonia - Which Policy Is To Be Preferred	1998
Reid M Ness, Ann M Holmes, Robert Klein, Robert Dittus	Cost-Utility Of One-Time Colonoscopic Screening For Colorectal Cancer At Various Ages	2000
Sujha Subramanian, Georgiy Bobashev, Robert J Morris	Modeling The Cost-Effectiveness Of Colorectal Cancer Screening Policy Guidance Based On Patient Preferences And Compliance	2009
Megan Smith, Jie Bin Lew, Kate Simms,Karen Canfell	Impact Of Hpv Sample Self-Collection For Underscreened Women In The Renewed Cervical Screening Program	2017
Parmigiani G, Skates S, Zelen M	Modeling And Optimization In Early Detection Programs With A Single Exam	2002
Cesare Hassan, Douglas K Rex,Angelo Zullo, Gregory S Cooper	Loss Of Efficacy And Cost-Effectiveness When Screening Colonoscopy Is Performed By Nongastroenterologists	2012
Mandelblatt J, Schechter Cb, Lawrence W, Yi B, Cullen J	The Spectrum Population Model Of The Impact Of Screening And Treatment On Us Breast Cancer Trends From 1975 To 2000 Principles And Practice Of The Model Methods	2006
Diaz M, De Sanjose S, Ortendahl J, Oshea M, Goldie Sj, Bosch Fx, Kim Jj	Cost-Effectiveness Of Human Papillomavirus Vaccination And Screening In Spain	2010
Berchi C, Bouvier V, Reaud Jm, Launoy G	Cost-Effectiveness Analysis Of Two Strategies For Mass Screening For Colorectal Cancer In France	2004
Guerrero Am, Genuino Aj, Santillan M, Praditsitthikorn N, Chantarastapornchit V, Teerawattananon Y, Alejandria M, Toral Ja	A Cost-Utility Analysis Of Cervical Cancer Screening And Human Papillomavirus Vaccination In The Philippines	2015
Megan A Smith, Dorota Gertig, Michaela Hall, Kate Simms, Jie-Bin Lew, Michael Malloy,Marion Saville, Karen Canfell,	Transitioning From Cytology-Based Screening To Hpv-Based Screening At Longer Intervals Implications For Resource Use	2016
Szucs Td, Largeron N, Dedes Kj, Rafia R, Benard S	Cost-Effectiveness Analysis Of Adding A Quadrivalent Hpv Vaccine To The Cervical Cancer Screening Programme In Switzerland	2008
Simon Lucas Goede, Linda Rabeneck, Iris Lansdorp-Vogelaar, Ann G Zauber, Lawrence F Paszat,Jeffrey S Hoch, Jean He Yong, Frank Van Hees, Jill Tinmouth, Marjolein Van Ballegooijen	The Impact Of Stratifying By Family History In Colorectal Cancer Screening Programs	2015
Sigrid V Carlsson, Tiago M De Carvalho, Student Monique J Roobol, Jonas Hugosson, Anssi Auvinen, Maciej Kwiatkowski, Arnauld Villers, Marco Zappa, Vera Nelen, Alvaro Paez, James A Eastham, Hans Lilja,Harry J De Koning, Andrew J Vickers, Eveline A M Heijnsdijk	Estimating The Harms And Benefits Of Prostate Cancer Screening As Used In Common Practice Versus Recommended Good Practice A Microsimulation Screening Analysis	2016
Dorte Gyrd-Hansen, Jessogaard, Ole Kronborg	Colorectal Cancer Screening:Efficiency And Effectiveness	1998

Hosking Michael, Roberts Stephen, Uzsoy Reha, Joseph Talisa M	Investigating Interventions For Increasing Colorectal Cancer Screening Insights From A Simulation Model	2013
Robert J Connor, Rob Boer, Philip C Prorok, Douglas L Weed	Investigation Of Design And Bias Issues In Case-Control Studies Of Cancer Screening Using Microsimulation	2000
Giulia Carreras, Giuseppe Gorini, Eugenio Paci	Can A National Lung Cancer Screening Program In Combination With Smoking Cessation Policies Cause An Early Decrease In Tobacco Deaths In Italy	2012
Arthi Vijayaraghavan, Molly B Efrusy, Marie-Hlne Mayrand, Christopher Csantas, Patricia Goggin	Cost-Effectiveness Of High-Risk Human Papillomavirus Testing For Cervical Cancer Screening In Quebec Canada	2010
Hakan Holmberg, Per Carlsson, Owe Lofman,Eberhard Varenhorst	Economic Evaluation Of Screening For Prostate Cancer: A Randomized Population Based Programme During A 10-Year Period In Sweden	1998
Arveux P, Wait S, Schaffer P	Building A Model To Determine The Cost-Effectiveness Of Breast Cancer Screening In France	2003
Jie-Bin Lew, D James B St John, Xiang-Ming Xu, Marjolein J E Greuter, Michael Caruana, Dayna R Cenin, Emily He, Marion Saville, Paul Grogan,Veerle M H Coup, Karen Canfell	Long-Term Evaluation Of Benefits, Harms, And Cost-Effectiveness Of The National Bowel Cancer Screening Program In Australia: A Modelling Study	2017
Sankatsing Vd, Heijnsdijk Ea, Van Luijt Pa, Van Ravesteyn Nt, Fracheboud J, De Koning Hj	Cost-Effectiveness Of Digital Mammography Screening Before The Age Of 50 In The Netherlands	2015
Beate Sandera, William Wl Wong, Man Wah Yeung, Orges Ormanidhi,Karen Atkin, Joan Murphy, Murray Krahn, Shelley L Deeks,	The Cost-Utility Of Integrated Cervical Cancer Prevention Strategies In The Ontario Setting - Can We Do Better	2016
Summer S Han, S Ayca Erdogan, Iakovos Toumazis, Ann Leung,Sylvia K Plevritis	Evaluating The Impact Of Varied Compliance To Lung Cancer Screening Recommendations Using A Microsimulation Model	2015
Zimmermann Marita R, Vodicka Elisabeth, Babigumira Joseph B, Okech Timothy, Mugo Nelly, Sakr Samah, Garrison Louis, Chung Michael	Cost-Effectiveness Of Cervical Cancer Screening And Preventative Cryotherapy At Anhiv Treatment Clinic In Kenya	2017
Haug U, Knudsen Ab, Kuntz Km	How Should Individuals With A False-Positive Fecal Occult Blood Test For Colorectal Cancer Be Managed A Decision Analysis	2012
Tiago M De Carvalho, Eveline Am Heijnsdijk, Harry J De Koning	Estimating The Individual Benefit Of Immediate Treatment Or Active Surveillance For Prostate Cancer After Screen-Detection In Older 65 Men	2015
Arrospide A, Rue M, Van Ravesteyn Nt, Comas M, Soto-Gordoa M, Sarriugarte G, Mar J	Economic Evaluation Of The Breast Cancer Screening Programme In The Basque Country Retrospective Cost-Effectiveness And Budget Impact Analysis	2016
Hassan C, Pickhardt Pj, Laghi A, Zullo A, Kim Dh, Iafrate F, Di Giulio L, Morini S	Impact Of Whole-Body Ct Screening On The Cost-Effectiveness Of Ct Colonography	2009
Philip E Castle, Cosette M Wheeler, Nicole G Campos, Stephen Sy, Emily A Burger, Jane J Kim	Inefficiencies Of Over-Screening And Under-Screening For Cervical Cancer Prevention In The Us	2018
Carles M, Vilaprinyo E, Cots F, Gregori A, Pla R, Romn R, Sala M, Maci F, Castells X, Rue M	Cost-Effectiveness Of Early Detection Of Breast Cancer In Catalonia Spain	2011
De Kok Im, Van Rosmalen J, Dillner J, Arbyn M, Sasieni P, Iftner T, Van Ballegooijen M	Primary Screening For Human Papillomavirus Compared With Cytology Screening For Cervical Cancer In European Settings: Costeffectiveness Analysis Based On A Dutch Microsimulation Model	2011
Andrea C Villanti, Yiding Jiang, David B Abrams, Bruce S Pyenson	A Cost-Utility Analysis Of Lung Cancer Screening And The Additional Benefits Of Incorporating Smoking Cessation Interventions	2013
Mcmahon Pm, Kong Cy, Johnson Be, Weinstein Mc, Weeks Jc, Tramontano Ac, Cipriano Le, Bouzan C, Gazelle Gs	Chapter 9 The Mgh-Hms Lung Cancer Policy Model Tobacco Control Versus Screening	2012

Nt Van Ravesteyn, Eam Heijnsdijk, G Draisma, Hj De Koning	Prediction Of Higher Mortality Reduction For The Uk Breast Screening Frequency Trial A Model-Based Approach On Screening Intervals	2011
Dennis G Fryback, Natasha K Stout, Marjorie A Rosenberg, Amy Trentham-Dietz,Vipat Kuruchittham, Patrick L Remington	The Wisconsin Breast Cancer Epidemiology Simulation Model	2006
Kj Carter, F Castro, E Kessler, B Erickson	A Computer Model For The Study Of Breast Cancer	2003
Vacek Pm, Skelly Jm, Geller Bm	Breast Cancer Risk Assessment In Women Aged 70 And Older	2011
Margarita Posso, Misericrdia Carles, Montserrat Ru, Teresa Puig,Xavier Bonfill	Cost-Effectiveness Of Double Reading Versus Single Reading Of Mammograms In A Breast Cancer Screening Programme	2016
Franka Loeve, Martin L Brown, Rob Boer, Marjolein Van Ballegooijen, Gerritt J Van Oortmarssen, J Dik F Habbema	Endoscopic Colorectal Cancer Screening: A Cost-Saving Analysis	2000
Johnston K	Modelling The Future Costs Of Breast Screening	2001
Taylor P	Modelling The Impact Of Changes In Sensitivity On The Outcomes Of The Uk Breast Screening Programme	2010
Arantzazu Arrospide, Isabel Idigoras, Javier Mar, Harry De Koning, Miriam Van Der Meulen,Myriam Soto-Gordoa, Jose Miguel Martinez-Llorente, Isabel Portillo, Eunate Arana-Arri, Oliver Ibarrondo, Iris Lansdorp-Vogelaar	Cost-Effectiveness And Budget Impact Analyses Of A Colorectal Cancer Screening Programme In A High Adenoma Prevalence Scenario Using Miscan-Colon Microsimulation Model	2018
Whyte S, Chilcott J, Halloran S	Reappraisal Of The Options For Colorectal Cancer Screening In England	2012
Juan P Wisnivesky, Alvin I Mushlin, Nachum Sicherman, Claudia Henschke	The Cost-Effectiveness Of Low-Dose Ct Screening For Lung Cancer*Preliminary Results Of Baseline Screening	2003
Djenaba A Joseph, Reinier G S Meester, Ann G Zauber, Diane L Manninen, Linda Winges, Fred B Dong, Brandy Peaker, Marjolein Van Ballegooijen	Colorectal Cancer Screening Estimated Future Colonoscopy Need And Current Volume And Capacity	2016
M Elske Van Den Akker-Van Marle, Marjolein Van Ballegooijen,Gerrit J Van Oortmarssen, Rob Boer, J Dik F Habbema	Cost-Effectiveness Of Cervical Cancer Screening Comparison Of Screening Policies	2002
Natasha S Crowcroft, Jemila S Hamid, Shelley L Deeks, John Frank,	Human Papilloma Virus Vaccination Programs Reduce Health Inequity In Most Scenarios: A Simulation Study	2012
Irene O L Wong, Mphil Janice W H Tsang, Benjamin J Cowling, Gabriel M Leung	Optimizing Resource Allocation For Breast Cancer Prevention And Care Among Hong Kong Chinese Women	2012
Dm Parkin, P Tappenden, A H Olsen, J Patnick, P Sasieni	Predicting The Impact Of The Screening Programme For Colorectal Cancer In The Uk	2008
Pamela M Mcmahon, Chung Yin Kong, Colleen Bouzan,Milton C Weinstein, Lauren E Cipriano, Angela C Tramontano, Bruce E Johnson, Jane C Weeks, G Scott Gazelle,	Cost-Effectiveness Of Computed Tomography Screening For Lung Cancer In The United States	2011
Mccann J, Treasure P, Duffy S	Modelling The Impact Of Detecting And Treating Carcinoma In Situ In A Breast Screening Programme	2004
Reka Pataky, Zahra Ismail, Andrew J Coldman, Mark Elwood,Karen Gelmon, Lindsay Hedden, Greg Hislop, Lisa Kan,Bonnie Mccoy, Ivo A Olivotto, Stuart Peacock	Cost-Effectiveness Of Annual Versus Biennial Screening Mammography For Women With High Mammographic Breast Density	2014
Jiang H, Brown Pe, Walter Sd	Inference On Cancer Screening Exam Accuracy Using Population-Level Administrative Data	2015
