# Supplementary material for: Cancer screening simulation models: a state of the art review
Source: BMC Med Inform Decis Mak. 2021 Dec 20;21:359. doi: 10.1186/s12911-021-01713-5 (PMC8690438; doi:10.1186/s12911-021-01713-5)
Supplement: Supplementary file 4 — Additional file 4. Detailed information on validation. [file 12911_2021_1713_MOESM4_ESM.pdf]

Filename	Model type	Method	What was validated	Data for validation	Complications	Type
10.1177@0272989x177181	individual level	Direct Comparison	incidence, mortality	randomized controlled trials		
wong2007.pdf	cohort level	Direct Comparison	mortality	randomized controlled trials		
arnold2018.pdf	individual level	AdViSHE protocol	incidence, mortality	SEER, other models		
chen2011.pdf	cohort level	Direct Comparison	incidence			
chiu2017.pdf	cohort level	Fitting				Internal
pataky2014.pdf	individual level	Direct Comparison	incidence	registry data, other models		
chauvin2013.pdf	cohort level	Direct Comparison	mortality	randomized controlled trials		
han2017.pdf	individual level	Direct Comparison	incidence, mortality	randomized controlled trials		
lejeune2003.pdf	cohort level	Direct Comparison	mortality	randomized controlled trials		
10.1002@ijc.29136.pdf	individual level	Direct Comparison	incidence	SEER	Slight overprediction	
zai2014.pdf	other	Barlas method				Internal
376.pdf	individual level	Direct Comparison	incidence		Optimistic staging	
weedonfekjaer2010.pdf	cohort level	Direct Comparison	incidence	other models		
lang2011.pdf	other	Fitting	quality of life			Internal
accetta2010.pdf	individual level	Direct Comparison	incidence		Lower prevalence	
lansdorpvogelaar2009.pdf	individual level	Direct Comparison	incidence, mortality	randomized controlled trials		
seigneurin2016.pdf	individual level	Fitting	incidence			Internal
rutter2016.pdf	individual level	Direct Comparison	incidence	randomized controlled trials, other models		
draisma2009.pdf	individual level	Direct Comparison	incidence	SEER		
hsieh2002.pdf	cohort level	Fitting				Internal
draisma2003.pdf	individual level	Direct Comparison	incidence	randomized controlled trials		
siebert2005.pdf	cohort level	Direct Comparison	incidence, mortality	registry data		
weedonfekjr2008.pdf	other	Direct Comparison	incidence	other models		
bergeron2008.pdf	cohort level	Direct Comparison	incidence, mortality	randomized controlled trials		
vijayaraghavan2009.pdf	cohort level, other	Fitting	mortality			Internal
berg2009.pdf	cohort level	Fitting				Internal
berkhof2010.pdf	cohort level, individual le	Fitting	incidence, mortality, detection			Internal
meza2014.pdf	individual level	Direct Comparison	incidence, mortality	randomized controlled trials		
gunsoy2014.pdf	cohort level	Direct Comparison	incidence			
steen2015.pdf	individual level	Direct Comparison	incidence	screening program		
uuskla2013.pdf	DE	Direct Comparison	incidence, mortality, sexual activity			
nihms115777.pdf	individual level	Direct Comparison	incidence			
gulati2012.pdf	individual level	Direct Comparison	incidence, mortality, staging			
barnett2017.pdf	regression,cohort level	Direct Comparison	incidence, mortality	literature		
gomez2014.pdf	cohort level	Direct Comparison	incidence, mortality	literature		
tay2011.pdf	DE,cohort level	Direct Comparison	incidence	registry data		
jgi_05076.pdf	other	Fitting				Internal
palma2015.pdf	DE,other	Direct Comparison	incidence	randomized controlled trials		

matthijsse2015.pdf	regression,individual leve	Fitting				Internal
campbell2016.pdf	cohort level	Direct Comparison	mortality	registry data	Slight overprediction	
sheehan2017.pdf	individual level	Direct Comparison	mortality	randomized controlled trials		
jeong2013.pdf	individual level	Direct Comparison	incidence, mortality		Slight differences	
munoz2014.pdf	individual level	Direct Comparison	incidence, mortality	SEER, BSCS		
10.2307@40271855.pdf	individual level	Direct Comparison	incidence, mortality			
sharma2015.pdf	cohort level	Direct Comparison	incidence			
sherlawjohnson2004.pdf	cohort level	Direct Comparison	incidence			
blanks2004.pdf	regression	Direct Comparison	test results			Internal
gamboa2008.pdf	cohort level	Direct Comparison	incidence, mortality	cross-sectional studies		
yamamoto2011.pdf	cohort level	Direct Comparison	mortality	registry data		
lin2011.pdf	other	Direct Comparison	mortality	randomized controlled trials		
roberts2007.pdf	individual level	Direct Comparison	incidence, mortality	SEER		
tramontano2016.pdf	individual level	Validated before				
timmers2013.pdf	regression	Fitting				Internal
janes2005.pdf	regression	Cross-validation				Internal
mandelblatt2009.pdf	individual level	Cross-validation		other models		Internal
duffy2014.pdf	other	Validated before				
prevost1998.pdf	other	Structural				Internal
10.1016@j.bpg.2010.07.00	individual level	Not applicable				
berkhof2006.pdf	cohort level	Direct Comparison	incidence			
rogoza2009.pdf	cohort level	Direct Comparison	incidence			
mandelblatt2002.pdf	cohort level	Direct Comparison	incidence			
petry2013.pdf	other	Fitting				Internal
gocgun2015.pdf	cohort level	Direct Comparison	incidence, mortality	CNBSS, literature		
berhane2003.pdf	regression	Cross-validation				Internal
duffy2009.pdf	other	Cross-validation				Internal
konno2010.pdf	cohort level	Direct Comparison	incidence			
kulasingam2009.pdf	cohort level	Direct Comparison	incidence, mortality			
mclay2010.pdf	cohort level	Direct Comparison	incidence, mortality	other models		
nevalainen2017.pdf	regression	Direct Comparison	mortality	randomized controlled trials		
canfell2004.pdf	cohort level	Direct Comparison	incidence			
etzioni2008.pdf	individual level	Direct Comparison	sojourn, lead times	literature		
coldman2015.pdf	individual level	Direct Comparison		randomized controlled trials		
berkhof2013.pdf	individual level	Direct Comparison	incidence	GLOBOCAN		
loeve1999.pdf	individual level	Direct Comparison	incidence, mortality	screening data		
lee2018.pdf	DE	Direct Comparison	incidence	SEER		
kim2004.pdf	cohort level	Direct Comparison	incidence	other models		
chauvin2011.pdf	cohort level	Direct Comparison	mortality	randomized controlled trials		

steen2015.pdf	individual level	Direct Comparison	incidence	screening data		
nahvijou2016.pdf	cohort level	Direct Comparison	incidence, mortality	GLOBOCAN,literature		
kim2012.pdf	cohort level	Fitting				Internal
schillerfruehwirth2017.pdf	individual level	Direct Comparison	mortality	literature		
knudsen2012.pdf	individual level	Direct Comparison	incidence	SEER		
ness2000.pdf	cohort level	Direct Comparison	incidence	SEER, literature		
hassan2012.pdf	cohort level	Direct Comparison	incidence, mortality	SEER		
lgj008.pdf	individual level	Direct Comparison	incidence			
guerrero2015.pdf	cohort level	Direct Comparison	incidence	GLOBOCAN		
szucs2008.pdf	cohort level	Direct Comparison	incidence, mortality			
gyrdhansen1998.pdf	cohort level	Direct Comparison	mortality	other models		
hosking2013.pdf	DE	Fitting				Internal
carreras2012.pdf	DE	Validated before				
10.2307@41995445.pdf	cohort level	Direct Comparison	incidence	literature		
arveux2003.pdf	cohort level			screening data		
sankatsing2015.pdf	individual level	Direct Comparison	incidence, mortality	screening data		
sander2016.pdf	DE	Validated before				
han2017 (1).pdf	individual level	Direct Comparison	incidence, mortality	NHS, NLST		
hassan2009.pdf	cohort level	Direct Comparison	incidence, mortality	SEER		
nihms408197.pdf	individual level	Direct Comparison	incidence, mortality	SEER		
10.1038@bjc.2011.300.pdf	individual level	Direct Comparison	incidence	NHSBSP		
lgj007.pdf	individual level	Direct Comparison	incidence	SEER		
carter2003.pdf	cohort level	Direct Comparison	mortality	NCDB, screening data		
arrospide2018.pdf	individual level	Direct Comparison	prevalence	COLONPREV		
whyte2012.pdf	cohort level	Direct Comparison	incidence	National Cancer Data Repository		
crowcroft2012.pdf	regression	Wasn't validated				
mcmahon2011.pdf	individual level	Direct Comparison	incidence, mortality			
mccann2004.pdf	regression	Fitting	incidence			Internal
pataky2014 (1).pdf	cohort level	Validated before				
